# Dynamic and static circulating cancer microRNA biomarkers – a validation study

**DOI:** 10.1080/15476286.2022.2154470

**Published:** 2022-12-13

**Authors:** Masood Abu-Halima, Andreas Keller, Lea Simone Becker, Ulrike Fischer, Annika Engel, Nicole Ludwig, Fabian Kern, Trine B. Rounge, Hilde Langseth, Eckart Meese, Verena Keller

**Affiliations:** a Institute of Human Genetics, Saarland University, Homburg, Germany; b These authors contributed equally to the study; c Helmholtz Institute for Pharmaceutical Research Saarland (HIPS), Helmholtz Center for Infection Research, Saarland University Campus, Saarbrücken, Germany; d Helmholtz Institute for Pharmaceutical Research Saar, Saarbrücken, Germany; e Department of Research, Cancer Registry of Norway, Norway; f Centre for Bioinformatics, Department of Pharmacy, University of Oslo, Norway; g Department of Internal Medicine, Saarland University, Homburg, Germany; h Internal Medicine, Saarland University, Homburg, Germany

**Keywords:** microRNA, cancer, colon cancer, breast cancer, biomarker, miR-99a, miR-155

## Abstract

For cancers and other pathologies, early diagnosis remains the most promising path to survival. Profiling of longitudinal cohorts facilitates insights into trajectories of biomarkers. We measured microRNA expression in 240 serum samples from patients with colon, lung, and breast cancer and from cancer-free controls. Each patient provided at least two serum samples, one prior to diagnosis and one following diagnosis. The median time interval between the samples was 11.6 years. Using computational models, we evaluated the circulating profiles of 21 microRNAs. The analysis yielded two sets of biomarkers, static ones that show an absolute difference between certain cancer types and controls and dynamic ones where the level over time provided higher diagnostic information content. In the first group, miR-99a-5p stands out for all three cancer types. In the second group, miR-155-5p allows to predict lung cancers and colon cancers. Classification in samples from cancer and non-cancer patients using gradient boosted trees reached an average accuracy of 79.9%. The results suggest that individual change over time or an absolute value at one time point may predict a disease with high specificity and sensitivity.

## Introduction

Noncoding RNAs (ncRNAs) regulated physiological and pathological functions. One regulatory mechanism relies on base pairing between coding and non-coding RNAs in protein complexes [1]. microRNAs (miRNAs) belong to such master regulators. Reported first in *Caenorhabditis elegans* [2,3], researchers discovered over three decades how miRNAs are transcribed, processed and repress genes [4]. The molecules are collected and systematically annotated in different databases, (including the reference database miRBase [5], MirGeneDB [6,7] or miRCarta [8]). MicroRNAs and isoforms of microRNAs are expressed in a very tissue- and cell type-specific manner or with a specific disease [9–14]. Several studies describe their potential as biomarkers [15–20]. While some studies rely on smaller cohorts, more recent research also includes larger case-control studies or longitudinal measurement of circulating microRNAs as biomarkers [21–23]. In longitudinal studies, the individual changes of markers instead of the absolute value can be considered. We previously analysed data from the Norway-based Janus Serum Bank, demonstrating stable measurement of profiles decades ahead of the actual cancer diagnosis [24–28]. Based on the previous results, we defined a panel of 48 microRNAs that we measured for different cancer patients and controls prior to diagnosis to identify and validate potential cancer biomarkers. To ensure a high data quality we applied a stringent filtering and included 21 miRNAs with stable expression above the background in the analysis. For each patient and control sample, we ensured that at least one time point prior to the diagnosis was available to facilitate paired data analysis and to evaluate the potential of dynamic changes in miRNAs as biomarkers.

The aim of the present study was to validate previous identified miRNAs with their respect to detect cancer prior to the diagnosis.

## Results

### Pre- and post-cancer serum miRNA patterns characterize molecular disease trajectories

**Figure 1.**
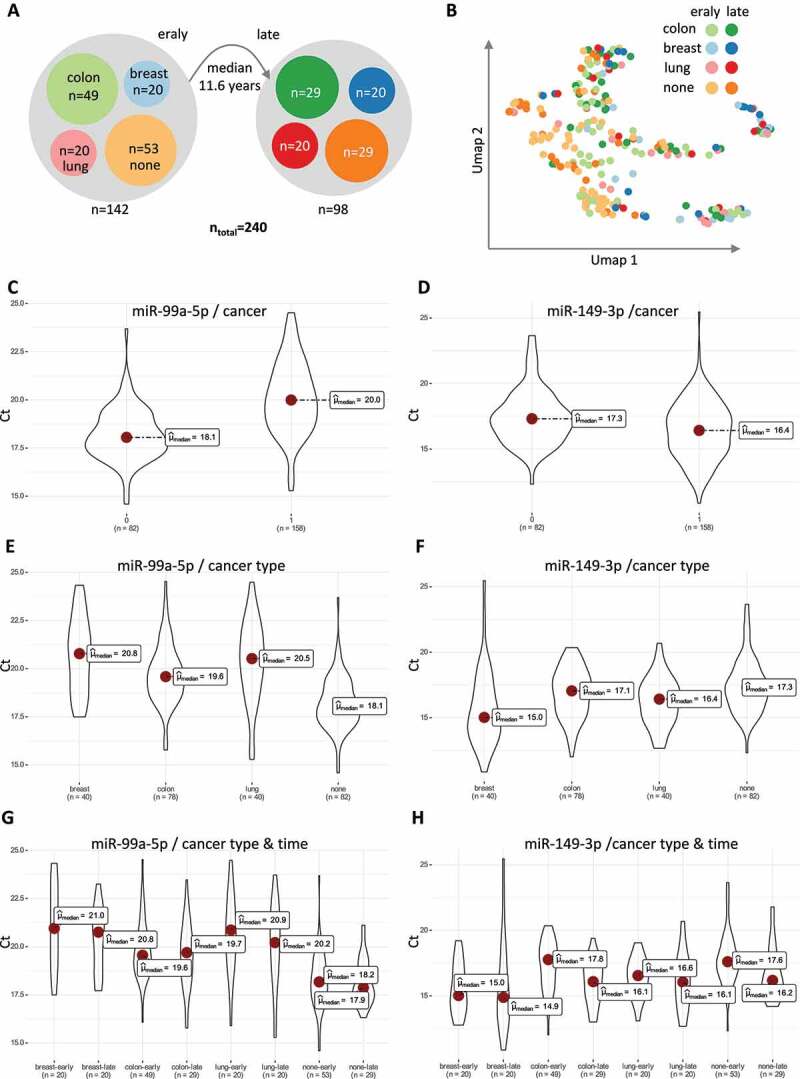
Study Set Up and Static biomarker analysis. **A**. The bubbles represent the different cohorts. The left bubble are pre-cancer samples and matched controls. The right bubble post-cancer samples and controls. The median time between the measurements is 11.6 years. Colours represent the different cancer types and controls. **B**. UMAP embedding for all 240 samples coloured with respect to the eight sub-cohorts. **C**. Beeswarm and Violin plots for miR-99a-5p for all samples from cancer patients (1) and controls (0). The Y-axis denotes the Ct value and the median for both groups is marked by a red dot. **D**. Same as panel C but for miR 149–3p. **E**. The cancer samples are split in the three different sub-cohorts for miR-99a-5p. **F**. Same as panel E but for miR-149-3p. **G**. The four cohorts are split up in the early time point and the late time point for miR-99a-3p. **H**. Same as panel G but for miR-149-3p.

We evaluated RT-qPCR biomarker profiles for three cancers (lung, breast, and colon cancer) and compared them to cancer-free controls. As mentioned, we have for all cancer cases at least one time point years prior to cancer diagnosis (***Supplemental Table 1***). The median time interval between the pre- and post-diagnostic samples exceeds one decade (Figure. 1A). Such measurements are only facilitated by substantial cohort studies, in our case the Norway-based Janus Serum Bank study [29]. Previously, we already described general patterns of biomarkers in this cohort and evaluated them with respect to their stability over time, indicating that we succeed to generate high-quality microRNA profiles independent from the age and storage time of samples in the Janus Serum Bank [30].

We included altogether 240 serum samples. For these, we initially generated profiles of 48 microRNAs in duplicates. A first stringent quality filtering reduced the number to 21 miRNAs, because all markers close to the background were excluded. The cohort of individuals split in pre- and post-diagnostic samples and highlights an intentional enrichment of colon cancer patients (Figure. 1A). Following previous results, we picked this cancer type as one of the most promising ones as an accurate diagnostic test. As a first view on the data, we performed a two-dimensional embedding (Figure. 1B). Such an embedding can highlight valuable biological information but also indicate potential technical issues. In our case, we note a tendency of control samples collected corresponding to the pre-diagnostic cancer time point to cluster together. Paired controls corresponding to the post-diagnostic cancer time point show similarities. Other clusters in the embedding are enriched for samples from cancer patients, independent whether the samples have been collected prior to or following the diagnosis. These results call for a more detailed consideration of single microRNAs and split in different case ontologies.

For each of the 21 microRNAs, we applied the same computational and statistical approaches to quantify the difference between the available groups, like we would do it in standard case-control study set-ups. The first aspect of our analysis was to test whether differences between cancer patients and controls independent of the actual clinical manifestation and a positive diagnosis exists. We computed significance values using a rank-based test because not all miRNAs were normally distributed. For each miRNA, we obtained one significance value for the Ct values of all samples from the cancer patients versus Ct values of all samples from the cancer-free controls. Most significantly, miR-99a-5p reached a p-value of 10^−15^ (Kruskal–Wallis test) and was 3.7-fold higher expressed in controls (Figure. 1B). In the other direction, miR-149-3p reached a p-value of 10^−3^ and was 1.9-fold higher expressed in serum samples of cancer patients (Figure. 1C). For the latter miRNA (that was also among the top candidates in our previous Janus studies), we spotted differences in expression between the different cancer entities. We thus repeated the above calculations but split the cancer cases in three sub-groups, colon, lung, and breast cancer (Figure. 1D).

For miR-99a-5p, Ct values of all cancer types consistently showed downregulation (Figure. 1E). But for miR-149-3p we note a substantial difference between the considered cancer types. Between breast cancer and colon cancer we observe a 4.4-fold difference in expression based on the Ct values (Figure. 1F). We next compared the pre- and post-diagnostic samples of all cancers but ignore whether the samples were taken before or after the cancer diagnosis. In the last comparison, we split the four groups in the early- and late-time points. The time analysis confirms the finding for miR-99a-5p: while all samples from cancer samples are lower expressed than all samples from non-cancer patients. The differences between the early- and late-time samples are not significant (Figure. 1G). Supporting this, we also computed p-values between all early- and late-time points, not highlighting substantial difference between them. miR-149-3p was expressed in similar levels for pre- and post-diagnostic samples of lung- and breast cancer patients. For colon cancer patient, the abundance varies by a factor of 3.2-fold between the pre- to the post-diagnostic time point, marking a significant up-regulation following the diagnosis. Of note, also the control cohort revealed an increase over time but to a lower amount (Figure. 1H).

Given the 21 microRNAs (features) and the 240 samples we can ask whether machine learning facilitates a classification in samples from cancer and non-cancer patients. We performed a fivefold cross validation, paying attention that samples of one individual are assigned always to the same split to limit a potential overtraining effect. Applying gradient boosted tree classification, we reached an averaged accuracy of 79.9%. Considering the details of the classification performance we find that the sensitivity (87.8%) exceeded the specificity (67.3%). Interestingly, the false positive classifications (controls predicted to suffer or to get cancer) and false negative classifications (samples from the cancer group assigned to the controls) split similar between the early and late time points. Considering the feature importance values, we obtained similar results to the single biomarker analysis. The features with highest importance values were miR-99a-5p, miR-186-5p, miR-140-5p and miR-484 (summing up to 40.9% of the overall feature importance).

Our results indicate differences in the trajectories of cancer biomarkers. While we focused on two prominent examples that represent different groups – either with stable expression over time or with changes over time. Multiple other markers in our study showed similar patterns, including miR-100-5p, miR-575, let-7d-3p. We thus provide all expression values for the 21 miRNAs in the 8 groups (***Supplemental Table 1)***. Especially, the change over time emphasizes how important longitudinal measurements are. We thus performed paired analyses and now consider the early to the late time point for each patient individually.

## Individual serum cancer markers are frequently not specific for one cancer type

**Figure 2.**
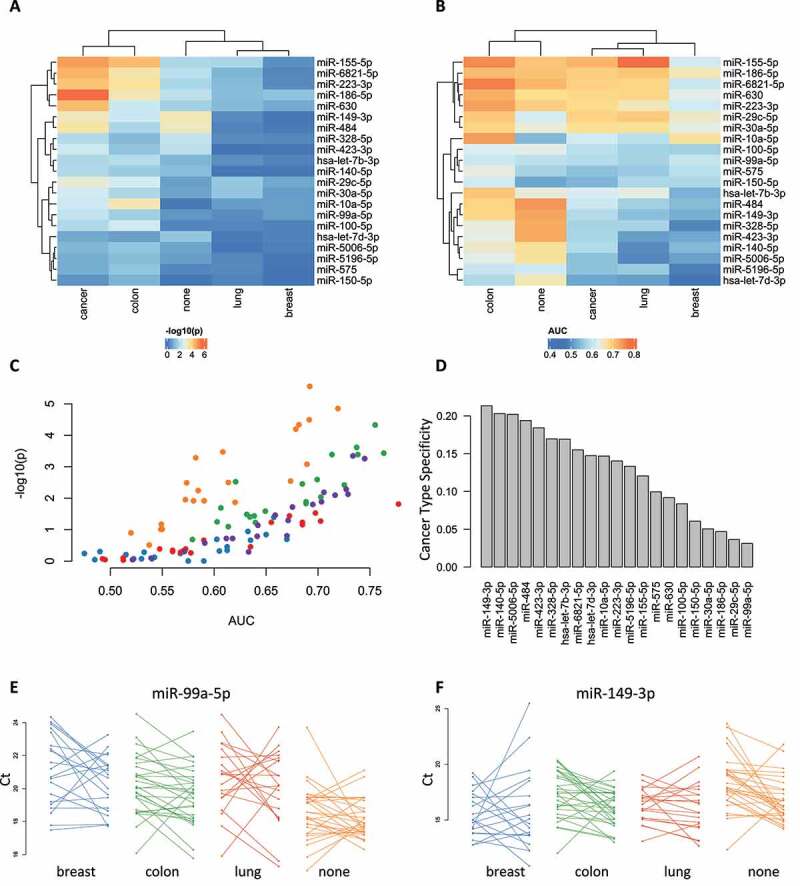
Dynamic biomarker analysis. **A**. Heatmap that displays the significance values for paired Wilcoxon-Mann Whitney tests between the early and late time point as negative decade logarithm for the 21 miRNAs and the 5 comparisons (three cancer types; controls; all cancers together). Dendrograms on top and right represent the clustering. **B**. Same as panel A but for the AUC values. **C**. AUC values versus p-values coloured by the cancer type and controls. The controls and colon cancer where most samples are included yield higher AUC values compared to the respective p-values. **D**. Specificity scores for the 21 miRNAs for one cancer. Left miRNAs are more specific as miRNAs on the right side. **E**. Line plot for miR-99a-5p that represent for each individual sample the Ct value of the early and late time point, connected by a line. The colours again represent the different cancer types and controls. **F**. Same as panel E but for miR-149-3p.

To identify early disease markers, we asked for miRNAs that are not significant for controls but significant for cancer cases prior to and at the time point corresponding to the cancer diagnosis. We thus performed pair-wise Wilcoxon-Mann-Whitney tests between the Ct values of all pre- and post-diagnostic samples and the corresponding Ct values from controls (Figure. 2A). For the controls, two miRNAs stand out by low significance values: miR-484 and the afore mentioned miR-149-3p. For breast and lung cancers, we found less significant effects. While several markers were nominally significant at an alpha level of 0.05 (e.g. miR-155-5p for lung cancer), they were not significant following adjustment for multiple testing. In contrast, for colon cancer multiple highly significant markers exist, such as the most significant miR-155-5p (p = 10^−^[5]). Likewise, we identified for cancers overall – and independent of the cancer type – highly significant markers. These however largely match the colon cancer markers. Because colon cancer is the group with most samples these general cancer markers are partially biased and dominated by colon cancer samples. This pattern altogether calls for caution with respect to p-values that are generally known. The decreased significance values for colon cancer samples and cancers might be inflated by the larger cohort sizes. Here, effect sizes are more appropriate than p-values. One commonly used measure for the performance of a biomarker is the area under the receiver operator characteristics curve (ROC curve), the so-called AUC value. The closer this value to 1, the better a biomarker separates two groups. Given the Ct values and the class labels, we computed the AUC values in the same manner for the same comparisons as we computed p-values (Figure. 2B). Then, we compared the p-values to the AUC values for the different cancer types (Figure. 2C).

In contrast to the p-value, we now also identify for the other cancer types markers with diagnostic potential, characterized by high AUC values. miR-155 again was among the most relevant markers but also miR-29c. The latter miRNA differed mostly between all cancer patients prior and post diagnosis. One remarkable finding is that miR-99a-5p, the most striking marker from the first part of our analysis, was the least notable one in the present analysis.

The type of biomarker, dynamic or static, might differ between different microRNAs. However, the specificity for certain cancer types might also vary, especially in conjunction with the afore mentioned differences. For each microRNA, we thus calculated the specificity for one cancer type, like a tissue specificity index. The specificity index can vary between 0 and 1, where a value of 0 represents a biomarker without any specificity and a value of 1 represents a biomarker for only one cancer type (Figure. 2D). The cancer specificity values for all microRNAs range between 0 and 0.25, i.e. the specificity for certain cancer types is generally limited. But especially, the markers that vary over time (miR-149-5p, miR-140-5p, miR-155-5p) have increased specificity values as compared to the other markers such as miR-99a-5p. The remarkable exception is miR-29c-5p that varies over time but is not specific for any of the cancer types.

The aggregated analyses suggest valuable biomarker profiles. A detailed inspection is required to verify that the results also hold on an individual basis. We thus computed line plots for the biomarkers where the early to the late time point for each cancer and each patient is connected. Likewise, the controls are displayed in the same manner. For miR-99a-5p, we obtained the expected results: samples from cancer patients have lower expression compared to samples from controls, independent of the time point (Figure. 2E). As second example, we present miR-149-3p (Figure. 2F). In this case, the miRNA is lower expressed in controls as compared to cancer samples. Further, for colon cancer and controls, we monitor increasing expression over time while patterns for lung cancer and breast cancer are heterogenous. Here, a high level of the miRNA together with a further increase over time can be predictive for colon cancer. But, it is important to mention that controls show similar patterns as colon cancer samples (false positives) and colon cancer patients like controls (false negatives).

From the results obtained herein, we derive three important conclusions. First, in-line with other studies, we note the limited value of p-values or at least the importance to consider both, p-values and effect sizes. Second, also matching other studies, we conclude that significant effects between cohorts still do not necessarily allow to perform accurate diagnoses on an individual level. Third, it is important to understand that biomarkers can follow different trajectories, i.e. they might be static and already dysregulated long before a diagnosis (comparable to genetic markers) or they might be dynamic and show an increase or decrease over time. These factors should be included in current biomarker studies. It is important to mention that case-control studies have the potential to discover both types of biomarkers, but it is not feasible to understand to which group the markers belong.

## Biomarkers split in static and dynamic markers

**Figure 3.**
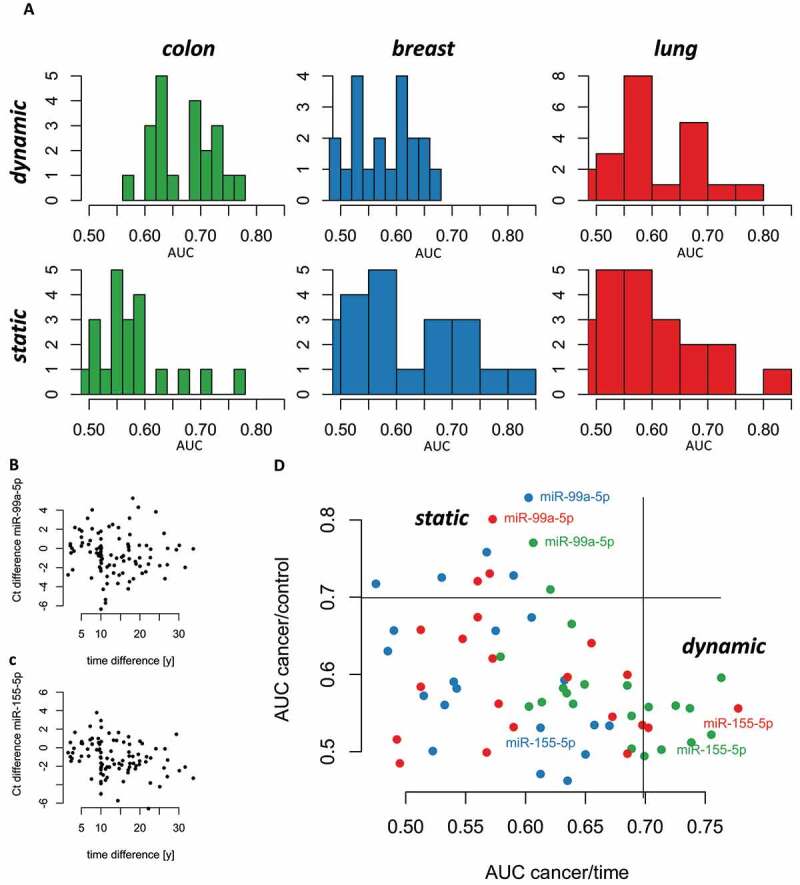
Dynamic versus static biomarkers. **A**. Histogram plots for the AUC values of cancer comparisons either as static markers (versus controls) or as dynamic markers (early versus late time points). **B**. Scatter plot showing the distance between measurements for each individual versus the difference in the Ct values for miR-99a-5p. **C**. Same as B but for miR-155-5p. **D**. Scatter plot of the static AUC values versus the dynamic ones coloured by the cancer type. The area on the right represents dynamic markers, the area on top static markers. No biomarker has high AUC values for both scenarios.

Our analyses suggest a split of biomarkers in static ones that are dysregulated at least a decade prior to the cancer diagnosis and dynamic ones that vary over time. The first group of markers is less specific for one cancer type as the second group. To substantiate the findings and to group miRNAs in the three groups (static markers, dynamic markers, and no markers), we compared AUC values for both, the setting from the first stage of the analysis (Figure. 1, static) and the second stage (Figure. 2, dynamic). For the three cancer types, the pattern differs significantly (Figure. 3A; Kruskal Wallis test p-value < 10^−^[5]). For colon cancer, we observed a significant shift towards dynamic markers (paired Wilcoxon-Mann-Whitney test p-value of 0.0008). For the other cancers, we found a slight but not significant shift towards static markers (paired Wilcoxon-Mann-Whitney test p-value > 0.05). Even though we checked in our first analysis for significant changes between the early and late time points for miR-99a-5p we correlated the difference between the time point in years with the difference in the expression identified by Ct values. Our analysis suggests a limited influence of the age on the expression of the selected 21 miRNAs (Figure. 3B for miR-99a-5p; Figure. 3C for miR-155-5p).

Based on the histogram plots, we select a threshold of 0.7 to identify biomarkers with sufficient diagnostic potential. Correlating all static miRNA AUCs to all dynamic miRNA AUCs for the three cancer types identifies the three groups of markers (Figure. 3D). The lower left part of the scatter plot contains markers with limited diagnostic value. In the lower right part, we observe the dynamic markers that are enriched for colon cancer. In this area, we for example observe miR-155-5p for colon cancer and lung cancer while the miR-155-5p for breast cancer is in the not relevant area. In the upper left part of the scatter plot, representing static markers, we find miR-99a-5p for all three cancer types. No microRNA achieved high AUC values for the static and dynamic consideration, allowing for a clear separation between the two marker types.

## Discussion

In the present study, we compared pre- to post diagnostic markers in three cancer types and cancer-free controls. The markers were selected from our previous studies and the literature. As read out, we intentionally choose RT-qPCR compared to our previous studies were we typically used microarrays or NGS. Not all markers from the previous studies were confirmed, which might be due to technical biases inherent to the different profiling platforms [31]. But, our analysis yields a split of circulating serum biomarkers for cancer in two groups: static markers that are changed already years prior to diagnosis and dynamic markers that are changing over time. To be able to distinguish between these two groups are likely of high importance in diagnostic applications.

To investigate such questions, broad population-based studies such as the Janus Serum Bank study from Norway are required. But with such studies, also potential challenges arise. The storage time and condition of the samples varies for example tremendously. In the previous studies, we carefully checked respective confounding variables and demonstrated that the storage time has only a limited influence [24–28]. Other confounding variables such as the age [32], sex [33], ethnicity [34], treatment [35], physical fitness [36], even the season when samples were collected [37], and many others can affect biomarker signatures. From respective population-based studies typically only a subset of respective variable is available, further emphasizing the need for validation studies and for larger cohorts [23]. For many markers in the study, such as miR-99a-5p, miR-100, miR-150 (Supplemental Figure 1) and others, no significant difference in the Ct values between the early and late time points exists. For those miRNAs where significant differences exist (e.g. miR-155-5p) the absolute differences remained limited (Supplemental Figure 2). Taken together, our previous studies and the present results argue against a substantial influence of the storage time on the 21 biomarkers.

To account for technical effects, we followed a strict blinding scheme. The laboratory only got numbered samples without any class information or annotation and only after handing in the measured Ct values the class labels and annotation of the samples were provided for the statistical analysis. Despite this careful handling and cross-checking technical bias cannot be fully excluded in such studies that run over half a century. It is thus also mandatory to cross check the results with the literature and to gain insight in the biological effects and functionality of the biomarkers. In the present study, we included 21 miRNAs. One way is to apply miRNA set enrichment analysis, either using subsets or ordered lists of miRNAs. To this end, tools as TAM 2.0 [38], sTAM [39] or miEAA [40] exist. Respective analysis using the different tools and different analysis methods result in an enrichment of various diseases, including many cancers and also those cancers included in the study. But, the relevance of these analyses remains limited because we pre-selected the miRNAs with respect to their role in the respective diseases. In the light of this bias, complex functional analyses seem to be more appropriate for studies using high-throughput technologies such as next-generation sequencing. Targeted validation studies call for a more specific and manual analysis. We focused in the results on three cases with substantial effect sizes. miR-99a-5p as general static cancer marker, and miR-155-5p as well as miR-149-3p as dynamic marker with different cancer specificity scores. For miR-99a-5p PubMed (queried September 2022) lists 63 manuscripts with relevance to different cancer types, tissue specimens and measured with different technologies. Of note, most studies list an increased level of this miRNA in serum (and especially exosomes) [41–43] and a decreased value in tissue biopsies [44]. But for breast cancer, Du and co-workers report a significant downregulation in serum [44]. The literature is partially contradicting itself and because of the large heterogeneity from a clinical and experimental side it is hard to conclude on the validity. For miR-155-5p, we identified 237 hits in PubMed (queried September 2022) and for miR-149-3p 70 manuscripts. Comparing the results and conclusions in these manuscripts highlights a similarly complex landscape. One factor that adds to the complexity are microRNA isoforms, slightly modified molecules that can have different biological functions. Such variations can influence miRNA target genes in reporter assay experiments [45]. For several miRNAs similar effects are reported, including miR-31 isoforms [46], miR-222 isoforms [47] and miR-101 isoform [48]. Characterization of isomiRs e.g. by RT-qPCR can be challenging itself [49], adding to potentially blurred profiles in biomarker studies. All these findings are well in line with our previous review on the literature, underlining that diagnostic miRNA biomarkers have certainly a potential, but the associated challenges persist [50].

But in sum, longitudinal measurement and large cohorts facilitate an improved understanding of the potential of microRNA biomarkers. From our view, it is mandatory to distinguish between markers that are present ahead of the diagnosis and stable and markers that are dynamic and change over time. The dynamic levels of miRNA are shown in other cancer such as lung cancer [51] and testicular cancer [52]. Of note, markers from both groups can be suited as early diagnostic markers, but in the first case the absolute value is more important while in the second case the change of the marker over time has higher diagnostic potential. An improved understanding of the regulatory mechanisms can then lead to novel therapeutic strategies [53].

## Methods

### Patients and samples

The study participants have given a broad consent for their samples to be used in cancer research. The study is approved by the Norwegian regional committee for medical and health research ethics (REC no: 2013/614). Serum samples were collected over at least two different time points, i.e. one pre-diagnostic and one post-diagnostic time point. For the pre-diagnostics time point, a total of 142 samples from three different types of cancer including lung (n = 49), colon (n = 20), and breast cancer (n = 20) patients, and age and sex-matched cancer-free donors served as controls (n = 53), were included. For the post-diagnostic time point, a total of 98 samples including lung (n = 29), colon (n = 20), and breast cancer (n = 20) patients, and age and sex-matched healthy donors served as controls (n = 29), were included. All serum samples were stored at −25°C until RNA, including miRNA, was isolated. More details on the storage and sampling conditions are provided in our previous publications [24–28].

### RNA extraction

Total RNA, including miRNA, was isolated from 200 µl serum samples using the miRNeasy Serum/Plasma kit (Qiagen) as previously described (Keller et al., 2017, RNA biology). Using the QIAcube Robotic Workstation (Qiagen), RNA, including miRNAs were eluted in a final volume of 14 mL RNase-free water according to the manufacturer’s recommendations. Small RNA Analysis Kit on the 2100 Bioanalyzer (Agilent Technologies) was used to resolve and quantify the small nucleic acid fraction of extracted total RNAs and concentration and purity were measured using NanoDrop™ 2000c Spectrophotometers (Thermo Fisher Scientific).

### RT-qPCR measurement

The expression level of 48 miRNAs was quantified by RT-qPCR using the Biomark™ HD system (Fluidigm Corporation). These 48 miRNAs were chosen based on their differential expression level in patient group compared to matched controls, as determined previously and based on their known associations with different types of cancer from the literature. TaqMan® MicroRNA Reverse Transcription Kit and RT Primer Pools (10X) (Thermo Fisher Scientific) were used. All steps were carried out according to the manufacturer’s recommendations. Briefly, 75 ng of RNA, including miRNAs were reverse transcribed into cDNA. The generated cDNA was pre-amplified using the TaqMan™ PreAmp Master Mix (2X) and the PreAmp Primers Pool (10X). Lastly, qPCR was performed using 96.96 Dynamic Array™ IFC (Fluidigm corporation). For every 10X Assay, 3 µl TaqMan Primer Assay (20X) (Thermo Fisher Scientific) and 3 µl Assay Loading Reagent (2X) (Fluidigm) were mixed, and a Sample Pre-Mix was prepared by combining 3 μl TaqMan™ Universal PCR Master Mix, no AmpErase™ UNG (2X) (Thermo Fisher Scientific), 0.3 μl GE Sample Loading Reagent (20X) (Fluidigm corporation) and 2.7 μl pre-amplified cDNA for each sample. The array was loaded such that 5 µl of the Assay Mix and 5 µl of the Sample mixture were added to each inlet and placed in the Biomark™ HD system. For quantification, the GT 96 × 96 Standard v1 PCR thermal protocol was used.

### Data analysis

Each of the 48 miRNAs was measured in duplicates, and the average of the expression was computed. miRNAs with more than 10 not available measurements among the 240 samples (~4% missing values per miRNA) were removed as lowly expressed or technically instable from further considerations. For the remaining miRNAs missing values were replaced by the global mean of that miRNA. 21 miRNAs passed that filtering step and were included in the analysis. All subsequent analyses have been carried out with R version 4.1.2.

2D embedding has been performed using the umap function from the umap package and the two coordinates are shown as scatter plots. Because not all miRNAs are normally distributed, we carried out non-parametric rank-based statistical tests if not mentioned explicitly (Kruskal Wallis Tests and Wilcoxon-Mann Whitney tests either in a paired or non-paired manner depending on the hypothesis). Reported p-values are nominal but we also checked whether p-values are significant following adjustment for multiple testing (Benjamini-Hochberg). As alpha level, we used the 0.05 threshold. The area under the receiver operator characteristics curve (AUC value) was calculated using the pROC package. Hierarchical clustering was performed using the Heatmap function from the ComplexHeatmap package. Beeswarm and volin plotes are generated using the ggstatsplot package with the function ggbetweenstats. Histograms are computed using the hist function with 10 classes.

## Supplementary Material

Supplemental Material

## Data Availability

All data are available in Supplemental Table 1 of this manuscript.
