# Supplementary material for: Dynamic and static circulating cancer microRNA biomarkers – a validation study
Source: RNA Biol. 2022 Dec 13;20(1):1–9. doi: 10.1080/15476286.2022.2154470 (PMC9754110; doi:10.1080/15476286.2022.2154470)
Supplement: Supplemental Material [file KRNB_A_2154470_SM8738.pdf]

Cacostat	1	1	1	1	0
PID	2071001	2071001	2071002	2071002	2071003
Date.of.blood.draw	15.01.08	15.02.91	15.05.01	15.04.91	15.04.88
Date.of.diagnosis	15. Jun 07	15. Jun 07	15. Sep 00	15. Sep 00	NA
type	colon	colon	breast	breast	none
hsa-let-7b-3p	18,5363655	17,9208855	20,8179756	19,6118422	19,5259552
hsa-let-7d-3p	14,1361727	16,5191342	17,5864855	12,9932732	18,6780238
miR-100-5p	16,7656681	16,8469308	15,960032	15,1633033	16,6089552
miR-10a-5p	17,1652866	18,9013614	19,4393296	18,1303942	19,3343179
miR-140-5p	13,4008878	15,5007326	14,4114608	12,1318329	18,4002889
miR-149-3p	13,4102962	15,2201239	16,2960417	14,7825578	19,6390873
miR-150-5p	8,75217069	9,99772419	11,4139848	9,74836721	10,846327
miR-155-5p	14,5341931	17,2386717	16,7897917	14,5982163	15,6342169
miR-186-5p	16,1384971	19,9051142	15,7286166	16,2362049	21,7444944
miR-223-3p	4,4594546	6,4081598	7,1446197	6,6606012	8,7420684
miR-29c-5p	14,1113384	16,1096434	15,4391988	15,4455241	17,2869258
miR-30a-5p	10,442682	11,8810596	11,8845636	9,57114151	11,9227939
miR-328-5p	13,6155095	14,9341922	15,0266815	13,4023466	17,3515232
miR-423-3p	20,5576868	21,446792	22,4681423	19,2915413	25,1328184
miR-484	11,0408988	13,1076146	12,8833827	7,93528663	13,8066063
miR-5006-5p	9,9824064	11,0725719	12,080762	10,9246614	14,6220167
miR-5196-5p	20,4250037	21,0159558	21,5422485	17,3735871	24,7384087
miR-575	9,69173421	11,1000393	12,5250461	10,6755319	12,8789736
miR-630	13,6261666	17,485077	17,2016355	14,872425	21,7833883
miR-6821-5p	8,58600071	11,0078584	11,8657191	8,6865126	14,8812256
miR-99a-5p	18,9843831	18,5348947	17,8251379	17,7738837	17,5605794

0	0	1	1	0	0	1
2071003	2071003	2071004	2071004	2071005	2071005	2071006
15.09.81	15.09.76	15.05.00	15.12.90	15.12.81	15.09.74	15.07.01
NA	NA	15mar2000	15mar2000	NA	NA	15mar2001
none	none	colon	colon	none	none	colon
18,2455571	19,1002534	19,9716588	19,7693222	19,2526363	20,3250157	18,925959
16,9550176	17,4656085	15,8219273	15,3177358	16,8582057	12,8433007	16,0211009
16,7449023	17,8261364	19,0799118	20,2897374	17,4629967	17,8979492	16,523421
17,4605876	20,7814475	17,7969834	17,5859865	19,5274948	18,4125826	20,0352294
15,8096868	18,7556638	16,4895494	16,3706058	15,9349179	11,7770113	14,7381437
17,8244361	20,18059	13,3117709	14,2939367	16,9405376	12,3346617	15,679515
9,08301888	8,93432007	9,86339695	9,0427237	10,2913127	8,35216293	10,1734456
16,2529413	17,7255721	15,7087313	15,1742516	15,5857799	13,2720837	14,9571614
21,4067499	25,8220229	23,2532477	21,8094469	20,8078269	13,5661912	18,2253694
8,72223421	11,1336726	9,51664171	8,900589	7,58422442	3,76989357	6,28685206
16,2710152	16,7168859	17,6203426	17,7680663	17,1807704	12,8760777	15,7954262
10,4392292	11,3656126	12,979583	12,4471584	12,4109836	9,3686817	11,6460729
15,4902168	17,586246	16,5536027	15,0811188	14,5585038	12,1377205	14,9805375
22,7526319	27,006524	24,5247078	23,2444123	21,6903564	16,4654581	22,2317099
12,7011877	22,1730595	13,8262533	12,8376935	11,9898653	9,55554052	13,803968
11,8662853	12,2089025	12,0348116	11,7997507	12,034093	8,48954087	10,9858095
18,9281043	22,7945565	22,9579667	25,2326108	20,491991	17,3902341	20,674933
10,0908134	10,9854102	9,98692503	9,16526533	12,387453	8,86645927	11,4148875
19,049671	23,518394	20,1183196	20,0021646	19,8251221	9,5067869	16,9075311
12,6403358	16,4484175	13,1582846	12,6701497	12,5475894	5,97547496	11,2729374
19,0747261	20,486941	21,0783236	21,84033	19,2864782	19,6710051	19,1267873

1	1	1	1	1	1	1
2071006	2071008	2071008	2071010	2071010	2071011	2071011
15.02.88	15.05.08	15.09.74	15.08.00	15.02.86	15.06.90	15.04.88
15mar2001	15. Apr 08	15. Apr 08	15. Feb 00	15. Feb 00	15may1990	15may1990
colon	colon	colon	breast	breast	lung	lung
19,0244306	18,4397501	20,3560948	23,3652423	20,2539631	19,1552573	19,973366
15,896944	12,8753668	16,6451078	18,9715634	16,8818083	17,8300899	15,8767221
18,1292536	17,6990123	18,7882499	20,3264174	17,1307038	17,3429494	17,9806942
18,7956144	17,163037	18,4237256	22,8117851	18,8506857	21,7239449	17,4944171
16,5568757	13,6304979	16,2640426	19,7353961	15,0703419	18,7342161	16,226225
15,9542277	13,0144044	17,3146112	22,3989121	17,586844	17,2666334	17,030865
10,5441589	8,67921217	9,56562685	17,7779721	11,2658425	10,5498359	9,2283
16,7384511	13,5801501	16,8781736	17,6937707	16,0591069	14,1902157	15,174037
20,3797572	18,2799186	21,115437	22,5442517	18,5579985	18,5338235	19,7300133
9,07476789	4,87080409	9,20205254	10,3791789	7,57194117	9,71134839	9,64689267
18,286069	14,0482301	17,1820633	22,3151891	16,2212382	14,0974833	15,204681
12,6317917	10,3718903	13,4398792	17,0347167	12,06588	10,3143323	11,5729999
15,888198	13,5752492	16,1093837	22,4417666	15,1421775	15,1345414	15,3112166
23,2105537	20,3528384	23,5773734	16,4578998	23,518237	24,5967477	24,7107003
14,1522127	11,760568	16,2306211	17,1498728	14,0110226	13,7020709	14,243946
12,6022388	9,13293221	11,57322	19,1391108	11,9799828	13,8790722	12,0516007
23,6434983	23,5142526	23,5170164	27,0438213	19,4614638	19,8684745	20,286544
11,7949319	8,25828955	11,0237882	18,6696594	12,3071576	11,8639297	10,3133532
19,2536698	14,3758318	19,9661855	19,2250586	18,0855632	18,3435499	20,0561862
13,2477333	8,90600447	13,3067434	15,4353615	12,9630558	12,5267825	13,6310903
20,8973119	20,7138867	20,7570293	21,2276591	19,0493303	20,1690341	19,8927923

0	0	0	1	1	0	0
2071012	2071012	2071012	2071013	2071013	2071015	2071015
15.01.88	15.01.83	15.01.78	15.11.98	15.09.86	15.10.86	15.10.81
NA	NA	NA	15oct1998	15oct1998	NA	NA
none	none	none	breast	breast	none	none
19,9948988	18,8218756	19,680161	18,6480299	19,6581171	19,443162	19,2099998
15,4204525	17,7182982	19,6372108	15,4420334	16,5018215	15,4057764	17,0747121
18,855179	17,0120719	17,4732805	16,2400387	18,0398441	16,6190759	17,7281711
19,5243733	21,505131	21,839281	17,8254301	23,9427101	17,554808	19,2898536
14,61926	16,4909506	20,2291327	15,3213152	17,0926567	15,1435299	15,9985133
14,965656	17,0844486	21,869504	17,1616229	17,0331851	15,9041169	18,1014787
10,8767441	11,189749	11,2811114	8,58629746	10,5277538	9,81033026	10,0487157
16,1426137	15,7325233	20,0157989	14,6198949	16,7197315	15,2157605	16,0749347
19,6014339	18,5775047	30,1810914	19,3902182	19,80241	18,9780763	20,0928028
6,67008218	8,09691696	9,97984024	8,46414312	7,29239194	6,31873432	7,62062684
15,5212635	16,6805838	19,5277711	15,4451456	16,7125093	15,5680095	15,2146677
11,5622632	11,3548962	13,399331	11,1084847	11,8832371	10,9903075	10,655819
14,667578	15,1312769	18,0936194	14,9043094	15,4955393	14,8035822	14,7518961
21,3399467	22,3653301	27,0544805	24,4131401	22,0630728	21,5077884	22,5158694
12,5944323	13,9278056	21,6944902	13,3688643	13,5169768	12,7683255	14,2085221
10,9647917	12,1643276	13,8794926	11,1619408	11,5922029	10,6441871	11,690933
20,9374215	18,6338942	25,4788868	18,92814	22,455244	19,4646682	18,691255
11,749761	13,4991857	14,2698409	10,8156263	12,4154162	10,9318866	11,7496069
17,1620077	17,917633	22,8673629	17,9292319	19,3061248	17,0714029	18,2737731
11,6081174	12,3114214	17,0090329	11,6242492	12,7289862	11,1709996	12,733882
21,1153242	19,0136371	19,3274171	18,6457308	20,3803723	18,8070764	19,7704471

0	1	1	1	1	0	0
2071015	2071016	2071016	2071017	2071017	2071018	2071018
15.10.76	15.08.06	15.07.89	15.10.02	15.01.87	15.10.86	15.09.81
NA	15may2006	15may2006	15. Sep 02	15. Sep 02	NA	NA
none	lung	lung	colon	colon	none	none
20,0701112	20,9553698	19,9719814	19,7549657	20,3527037	18,5112782	19,4907866
17,9888302	13,8687309	12,8909845	15,8554235	17,1119656	16,3912152	16,4870609
17,5421505	19,9865333	17,5242105	17,8072995	17,8904843	15,649209	16,9461898
18,9271622	17,4452625	17,3416286	18,1255358	20,6765007	17,1352964	18,6191433
16,9589378	16,4205283	14,8482002	14,9550762	17,3498428	15,8091943	16,6448285
19,1322516	14,660888	13,1040108	15,5652802	19,4693364	15,9223333	17,0373661
11,5088585	10,1284897	8,91643542	9,38730219	10,286761	9,85459877	10,7949355
17,5677932	15,7052647	14,5240217	13,6091477	17,2734709	14,2187178	16,6827416
24,6916599	21,8889398	18,0456137	16,9200127	21,6780906	17,721914	19,220589
9,21713324	7,83379672	6,01055353	5,91276727	8,17455426	6,38876098	7,47344611
18,1128772	18,0290556	16,4251953	15,1486146	16,3191473	14,9423927	16,5102673
12,7384823	12,6216878	11,0151943	11,2834898	11,9996658	11,2291216	11,5766208
18,2232613	15,764472	13,4878903	14,0486465	15,9324012	15,7849527	16,7742148
25,1946386	22,831696	20,6524898	22,0859023	22,7748998	21,5433643	21,6661954
18,7003196	13,8377017	10,7394451	12,2980034	14,8550927	13,1274028	12,6520178
13,2595146	11,8568311	9,82547984	10,729064	11,9842089	11,7426581	12,5424941
22,6103314	26,383611	25,2320812	19,2086769	21,418473	21,5019812	22,6811591
12,6423432	9,26547051	8,41597749	11,4491922	13,2597732	11,2693501	12,0824688
20,6426331	19,0368925	16,2351737	16,636366	19,7008145	17,2881952	19,4347701
15,5916349	11,9327783	9,54541252	11,121911	13,8342443	12,1363777	13,1227664
19,6561856	22,0156987	21,896437	19,6914763	20,4599412	16,8290397	17,9453595

0	1	1	1	1	1	1
2071018	2071019	2071019	2071019	2071019	2071020	2071020
15.10.76	15.10.99	15.12.86	15.01.82	15.01.77	15.09.95	15.09.81
NA	15. Nov 97	15. Nov 97	15. Nov 97	15. Nov 97	15. Aug 95	15. Aug 95
none	colon	colon	colon	colon	lung	lung
22,144407	19,4848504	18,6981185	19,7781546	22,2700091	19,9819131	20,5218004
17,9177443	16,4896284	17,9739053	17,8096031	17,3223621	20,5359077	16,1994512
16,9426994	18,084437	17,9830462	17,5693147	18,3912614	17,8555763	17,6350205
20,3407227	18,6692882	20,4182823	18,9632895	18,477067	23,9297361	17,5835004
17,590023	16,918858	17,3852053	16,1608788	17,784943	19,6219695	18,0567351
22,3534206	17,3166707	18,3803133	19,4396691	18,7643311	18,4140876	15,8235711
9,76583055	9,82971338	10,7354952	10,4973502	10,6746544	12,8097371	9,34362467
17,8859601	16,2096655	17,9067935	15,79788	17,3202981	16,0615024	15,2840891
25,5962558	19,049803	21,3549959	20,77328	25,9535477	21,5306058	21,7481367
10,9810935	6,82442586	8,50255482	8,38827161	9,58303915	9,58164293	9,55019884
17,5725249	15,1116141	16,5582319	17,1749722	16,5923688	16,0779621	17,0177318
13,1000705	11,1519937	11,6833591	12,1001479	13,5247179	12,3617881	11,6485612
17,079909	15,1879024	15,8261338	16,6224856	19,3465311	16,4677978	15,9293582
25,2470829	23,4881995	22,972578	25,8205577	27,7732778	28,2663326	23,7375996
18,8496693	12,7174334	14,4652716	15,530725	18,0454583	17,393229	13,5078436
13,6117363	11,7124262	12,3150206	13,0117798	13,4174102	15,1553649	12,26639
22,724215	21,1052219	20,1349378	21,2307019	20,9525094	19,8502902	25,1294065
12,2199548	11,9987193	11,9594526	12,4911661	12,4381852	14,9676508	10,2648461
23,7436278	17,8128021	19,2405771	20,3316683	21,6114532	22,8824832	19,5053286
16,2876023	11,1588421	13,7832361	14,3926456	15,3775611	16,1831636	13,2376842
19,1889978	19,9744708	19,9845674	20,0056367	20,0550483	19,896817	20,9134351

1	1	1	1	1	1	1
2071021	2071021	2071022	2071022	2071023	2071023	2071024
15.05.01	15.01.86	15.12.08	15.02.90	15.01.07	15.11.88	15.12.06
15mar2001	15mar2001	15. Jul 07	15. Jul 07	15dec2006	15dec2006	15oct2006
lung	lung	colon	colon	lung	lung	lung
20,09239	20,4218786	17,9599471	19,8129544	19,949943	19,3657351	22,024865
16,8728689	17,8869041	12,9192538	16,6042717	15,5674067	17,6135405	13,4031118
15,9453687	17,7963785	16,0910598	17,4818408	21,6140644	16,0894378	17,1827212
17,6114374	18,9391969	18,2306486	19,0250005	20,4036611	18,2258651	16,1982927
18,3091183	17,7244632	12,670878	16,7608111	18,5876741	16,7842096	13,4610394
17,257173	19,0539311	14,4880278	16,7729806	13,1166715	14,5148545	15,2291508
10,8689852	11,4573584	7,52126857	25,6584369	12,2308688	9,68806001	7,30340296
15,6475738	17,5160955	13,6751817	15,9627607	14,8652445	15,808326	14,110652
22,9061744	20,6872079	15,2802428	20,5236514	23,7821312	21,7298813	16,5748812
9,88606714	9,50019677	4,9716479	20,5939579	9,82420944	8,88595103	6,49920345
17,922557	16,5622583	14,3974199	16,7806806	18,5323649	17,2082362	13,2890541
12,5477952	12,434633	10,4991663	23,8975322	13,5087101	12,5417318	10,4247174
16,4573727	17,5060497	12,5457397	15,7857395	15,7315225	15,8190998	13,4981697
25,7937676	25,8067088	19,8974975	26,3475642	23,9786343	23,3140359	20,2570943
15,7300203	16,1327541	11,2805854	14,0622145	13,346154	14,2622926	11,776954
14,7316861	13,7574334	8,81796145	25,9920543	12,6115187	12,2054942	8,86734251
25,831701	22,6123035	18,3816943	22,3386964	27,6801978	22,2486108	20,0918
10,4388101	12,3313148	10,0368591	25,0333136	12,1381055	11,6137846	8,35836739
22,0337169	21,3706619	14,6187089	19,1598167	21,0149672	20,8872187	14,8423867
14,4954094	14,5368101	8,69061296	25,2749002	13,8477812	13,3873796	9,45830577
18,791948	19,7033745	18,0829453	19,4562995	23,7221974	18,4742084	18,2601484

1	1	1	1	1	1	1
2071024	2071025	2071025	2071025	2071025	2071027	2071027
15.08.88	15.03.04	15.01.82	15.07.76	15.11.74	15.02.08	15.01.91
15oct2006	15. Feb 04	15. Feb 04	15. Feb 04	15. Feb 04	15. Jul 02	15. Jul 02
lung	colon	colon	colon	colon	breast	breast
19,7031324	28,5110469	22,8298634	17,7963755	18,2766391	19,8129544	19,044728
14,6099377	18,5674236	15,3526468	16,1812625	13,7484986	24,4409526	15,1763809
19,6804037	17,9959505	16,8698573	17,4660241	17,1325816	17,4818408	17,2219583
17,271065	19,529054	18,6376335	22,7406235	18,5128995	19,0250005	18,4399971
17,0506005	15,147608	14,860282	16,6745318	11,9591162	24,6015626	14,8646592
15,896823	16,9722847	16,1082023	17,8554096	12,027318	25,4624613	14,3520735
9,46182368	11,4653391	10,6868542	11,879681	9,08856808	32,1478378	9,72246463
15,7985634	15,5426036	14,9552488	15,1805165	14,0907183	30,5359019	15,0569001
23,2686898	17,8445636	17,4618742	18,8572647	13,1446356	20,5236514	17,5897563
7,86335382	6,58087982	7,01023185	7,46143047	3,66279312	18,7621833	6,23478202
17,4472583	16,5738519	14,6307009	16,1500626	12,9335581	31,7823463	15,8107132
12,6567936	12,8516195	11,4032872	11,0660073	9,30148114	25,3606872	11,5535357
15,1099112	16,1894568	14,6588332	13,7541908	11,5400103	15,7857395	13,8681184
23,0489711	26,0650101	22,8969654	21,9773843	17,0299781	27,8983819	21,5522979
13,4813224	12,7084734	12,7749073	12,2838094	9,05492729	23,0354504	12,2110419
11,3922531	12,755922	10,8671369	11,3874544	8,86344092	25,4261722	10,5281706
27,1992285	20,5333721	18,8584949	21,4998495	17,508687	22,3386964	18,4844761
9,52185404	12,5178156	11,7308258	14,1793516	8,82727104	24,5219836	11,1157333
19,4819381	17,395452	16,2862569	18,2883561	10,4112047	38,2658933	15,8831328
12,3970024	11,8164296	11,3739214	11,8614433	6,20709491	22,9801068	10,7038105
21,4313473	19,4148124	19,1018284	20,180572	19,5537767	19,4562995	19,2049112

1	1	0	1	1	0	0
2071028	2071028	2071029	2071030	2071030	2071031	2071031
15.05.04	15.02.91	15.12.86	15.09.94	15.08.91	15.05.86	15.05.81
15. Apr 04	15. Apr 04	NA	15. Jun 94	15. Jun 94	NA	NA
breast	breast	none	colon	colon	none	none
19,7242956	19,6389355	19,1583136	18,9794658	19,8267706	19,8626378	19,4870556
15,6517648	13,3577632	16,460086	19,1212523	15,116651	20,6438839	17,5177991
17,9478361	20,2164378	15,8014065	16,3967934	16,2559292	16,7143105	16,0348017
17,8443744	17,3206563	18,5563894	18,1419207	16,3529753	21,04839	17,913261
16,9391922	15,7989735	16,4896991	17,5649682	14,6364462	18,1425443	16,6961187
15,451791	14,206802	15,3454172	19,3820089	16,1448757	21,7877437	17,5481969
10,1191031	8,94527582	10,7602652	10,0900683	7,93796854	11,9165536	10,3032522
16,0954156	15,289247	14,5499821	15,2324847	15,3099797	16,6253173	15,0216638
20,4382046	21,9345208	18,9220736	21,4401481	18,0223138	21,069877	19,088408
7,60631553	7,59851692	6,34889757	8,74014073	7,50382147	9,07861082	7,18573293
16,6074173	17,1345485	16,6283747	15,4984647	15,2380188	17,3228212	16,7641454
11,6577255	12,6564566	11,3810344	11,2795136	11,0017476	11,8512054	11,6779467
15,9431634	13,9283394	15,548859	15,7338643	14,9268695	17,5556054	16,4172004
24,102382	21,4655527	21,7438923	24,9643728	22,491185	23,2828063	22,9747622
13,4231778	12,8065599	12,0367145	15,1965749	13,0067482	14,583745	12,6671712
13,0249734	10,9910862	12,4649534	12,9739082	10,5082586	14,6787801	12,8516142
24,593628	26,669544	20,1086032	21,3595748	19,8313284	19,0883038	20,8509692
10,4693812	9,19358631	12,2337604	12,4054183	10,0846542	13,9372666	11,9831344
19,2237254	17,4469193	18,4593404	19,5830801	17,1067798	21,7215322	19,1591233
12,6179264	11,1612082	12,8683504	13,8426455	10,9122161	16,0930572	13,4035119
21,3450021	23,9099423	17,372348	18,1959743	18,479467	17,9150925	16,9309027

0	0	0	0	0	0	0
2071031	2071032	2071032	2071032	2071034	2071034	2071034
15.04.76	15.08.86	15.09.81	15.08.76	15.02.89	15.12.86	15.10.85
NA	NA	NA	NA	NA	NA	NA
none	none	none	none	none	none	none
19,8129544	19,0693227	20,4623303	18,3192371	19,8129544	23,9311143	20,768392
16,6042717	18,5044119	17,2764349	19,5106279	16,6042717	15,9677937	15,3419844
17,4818408	16,4753325	16,0729773	17,0200828	17,4818408	16,7805165	15,7821413
19,0250005	18,7786836	17,9724346	18,658325	19,0250005	17,9796687	19,2042138
16,7608111	17,3861633	17,8987813	17,7772824	16,7608111	16,4437927	15,9888985
16,7729806	18,9051366	18,9819365	19,3284526	16,7729806	16,3016605	16,309769
10,5533267	11,3706084	10,5178575	11,5536006	10,5533267	11,1170433	10,2015154
15,9627607	15,3812239	15,5357311	16,4869676	15,9627607	16,3681908	14,3104066
20,5236514	18,7864982	20,9822835	21,7292168	20,5236514	17,9966082	17,7386788
8,02432	7,64808297	7,30267022	9,01560557	8,02432	6,9576504	5,70398339
16,7806806	16,5437118	15,8526783	18,1676934	16,7806806	16,4593099	14,4450471
12,0438308	12,1587409	11,4333371	12,6868451	12,0438308	12,3249616	10,8100993
15,7857395	15,8513928	16,2185866	18,8722025	15,7857395	15,7344215	14,9241238
23,069167	22,985508	22,9311387	24,0207544	23,069167	20,1194727	20,6516327
14,0622145	13,4919972	12,9669921	17,33842	14,0622145	12,6254532	11,5077157
12,4886794	12,7852637	12,9726792	13,6367058	12,4886794	12,282721	11,4767985
22,3386964	19,7965462	23,4539937	22,5511651	22,3386964	23,3938778	20,4172897
11,7947995	12,9480859	12,0830853	13,3317014	11,7947995	12,1730485	11,6401064
19,1598167	19,926397	19,055013	21,3847016	19,1598167	18,1855761	16,682519
12,9938054	13,5102784	12,8325382	16,1954372	12,9938054	12,4831513	11,391754
19,4562995	17,4148254	17,4213067	18,274755	19,4562995	18,1998136	17,2676028

0	0	0	1	1	0	1
2071035	2071035	2071035	2071036	2071036	2071037	2071038
15.04.86	15.08.81	15.08.76	15.03.06	15.11.88	15.02.82	15.04.02
NA	NA	NA	15. Jan 06	15. Jan 06	NA	15. Jul 00
none	none	none	breast	breast	none	colon
18,4839344	20,2335209	20,2669666	24,4275711	20,801529	19,8129544	17,8477799
21,9183504	19,1692207	22,3892975	13,9651473	18,4888914	16,6042717	15,5744254
16,059386	16,1121695	16,2283737	18,414667	20,6717596	17,4818408	17,6961585
21,8023439	20,3094171	20,9092602	17,931189	21,6742718	19,0250005	18,1422737
24,6037285	20,2593672	22,6230151	15,7026344	17,0937731	16,7608111	16,477898
21,1826003	20,7844583	23,3137745	12,2739473	18,885729	16,7729806	15,0672373
12,6722897	11,5455921	10,9268316	8,3025183	9,84476047	10,5533267	9,11322086
16,6847508	16,4306149	16,8463262	14,1861769	16,2815776	15,9627607	14,7824118
26,7330578	25,2031148	25,3069952	20,300309	22,7489269	20,5236514	18,8684166
9,46303259	8,02438272	7,3328395	7,02939394	10,58027	8,02432	8,26730232
19,2132851	17,3113467	18,6106321	16,1473379	15,0895275	16,7806806	15,3025621
11,7222099	11,8901374	11,6961737	11,7132264	12,0638358	12,0438308	10,8660369
17,6706075	20,9609016	17,7528368	15,1361988	15,9097004	15,7857395	14,9879518
25,4076254	25,3590582	25,8130806	22,0565826	25,8422271	23,069167	22,602103
15,750625	17,0126212	20,8354487	12,0306218	15,8309088	14,0622145	12,1357956
15,9014333	14,5616183	13,989184	11,0656178	12,5573663	12,4886794	11,5824632
23,3147867	21,7594577	23,4956527	24,3287785	17,9930391	22,3386964	21,4771727
16,0549902	14,2386311	14,834551	9,50612041	12,9849106	11,7947995	10,2445271
26,4006573	21,6118525	24,9594583	17,5858612	21,7525075	19,1598167	17,8644944
16,9429229	16,4945824	16,7600496	10,1840286	15,0418987	12,9938054	11,3739909
17,3699013	17,5927313	17,273556	20,8535055	24,3290368	19,4562995	20,1768094

1	1	1	0	0	1	1
2071038	2071040	2071040	2071041	2071041	2071042	2071042
15.04.89	15.03.94	15.08.73	15.05.87	15.05.82	15.01.93	15.06.81
15. Jul 00	15. Feb 94	15. Feb 94	NA	NA	15. Jun 91	15. Jun 91
colon	colon	colon	none	none	lung	lung
21,1750537	19,1054869	19,893384	19,0958707	18,3247572	18,5352202	20,3233777
16,184881	18,5802128	18,7129397	17,1659449	16,8689062	17,7127271	17,0249595
16,0117521	18,7262958	22,1890895	16,430344	16,3654556	18,257513	21,4220492
18,446265	18,8161722	21,1369026	18,0422184	19,0036294	22,4335415	19,635959
15,3594424	23,4931331	20,7382777	16,0942721	15,3227249	17,4372839	18,8759724
15,1902642	17,9179745	19,4044527	15,4197667	15,4045323	17,7540534	17,983865
10,5151662	12,98252	11,9097815	10,8310555	10,8413226	11,3303761	11,0252341
16,0997696	19,0837836	18,6273612	15,0440269	15,9684182	15,4389632	18,6576225
20,6842116	29,7500897	31,0537216	18,7415345	18,8062052	18,3983007	24,5207018
7,21757916	13,0349273	14,4856739	6,80249044	7,03166462	8,51906266	10,8786632
16,3734256	21,2889025	21,9081148	14,8907901	17,5659353	15,6558283	20,0034588
12,7187073	13,6086973	15,588824	10,5859616	12,5544722	11,9477625	13,3875998
15,9507068	20,6637885	21,1223507	15,8554812	15,74035	15,6880633	16,6603914
22,7580042	23,069167	23,069167	21,0409227	20,0909031	24,9497624	24,6974781
13,7013979	18,1393874	14,0622145	13,2653754	12,6667551	14,0854115	15,3607149
11,5053592	18,0882075	16,947566	12,3214454	12,4560234	13,6719527	13,8694181
19,7158287	35,4423832	35,0018077	18,5307293	20,4652941	22,5434539	25,1783214
11,3821804	14,5839998	13,308088	11,8236481	12,6251869	13,4097845	11,4741938
18,4780393	27,3314081	27,4037801	18,2737971	18,363403	18,0427826	22,2875944
12,715598	17,9235374	20,4961876	12,7636148	12,2255827	11,5973873	15,1685254
17,678594	21,9234185	24,526533	17,398816	17,147713	20,7739846	22,7121137

1	1	1	1	1	1	1
2071044	2071044	2071044	2071044	2071045	2071045	2071046
15.03.03	15.02.88	15.02.83	15.02.78	15.04.01	15.08.86	15.02.03
15. Feb 03	15. Feb 03	15. Feb 03	15. Feb 03	15dec2000	15dec2000	15. Nov 00
colon	colon	colon	colon	lung	lung	colon
17,8194215	19,2073111	18,9508272	19,4318788	19,5458572	18,2468775	16,5068882
15,8755541	16,5558043	15,295499	19,1645911	16,8531343	16,8077742	14,054008
16,335245	16,844623	17,2097081	17,9651061	19,2758495	18,2856962	14,6204293
18,8743284	19,56595	19,606377	19,7206959	19,1176148	18,6564923	16,0045019
15,2501439	15,6333947	14,7837965	17,4422375	17,6166087	17,2969527	12,2219923
15,9102398	18,4039771	15,2667032	20,2126327	16,4458055	18,5641294	14,6627361
10,8098905	10,4884707	10,1525711	10,8940196	10,385382	10,407088	9,48203016
15,4832477	14,8135892	14,3604455	19,701692	16,0974791	17,8069681	12,0068547
17,0051989	19,9540625	16,6513142	22,7697562	25,2381265	23,5922072	15,5761992
6,28361011	7,68818087	6,48333327	8,92679781	8,38524539	10,337887	4,46461466
16,2957405	15,8947614	15,5257742	17,5922246	17,4456666	17,5588431	18,8623675
11,8594918	10,5723933	10,9919278	11,9874819	13,1129224	12,60103	14,9840466
14,0316062	14,6789916	13,985354	17,7203285	16,5875474	15,9253367	14,6235618
21,5123045	22,9610001	21,463452	24,8497553	25,300559	24,2664255	12,5254816
12,4414901	13,3747733	12,422434	19,0429326	16,0083515	16,0713992	11,4997596
10,9341231	11,7787574	10,5797487	12,7566901	14,5375571	13,4374985	10,0030562
19,7902827	18,9805768	17,8218963	23,1682304	28,7121986	19,2527881	18,752762
11,7331038	11,8227318	11,6340508	13,0125616	11,2267865	12,0598494	10,3702236
16,2658789	18,0332924	15,9385271	20,9662944	21,3011587	22,1429614	12,9792569
10,8551693	12,4982074	11,0693468	15,6521713	14,4492837	15,0202783	8,34844936
17,9225261	18,6974793	19,0836809	19,7203754	21,8647286	20,8552489	15,7794092

1	1	1	1	1	1	1
2071046	2071047	2071047	2071047	2071047	2071048	2071048
15.06.88	15.10.99	15.02.86	15.11.81	15.12.79	15.08.05	15.01.90
15. Nov 00	15. Sep 99	15. Sep 99	15. Sep 99	15. Sep 99	15. Jun 05	15. Jun 05
colon	colon	colon	colon	colon	breast	breast
19,4252682	18,4385994	21,8305883	21,6560874	22,437302	20,6440591	19,1068186
14,4956336	19,0485873	16,5674233	14,7164785	15,3499611	14,1729136	13,6444937
16,5446353	17,96912	18,4815483	16,9276002	17,862331	18,1586287	19,9304262
18,5192008	18,6952193	19,5915322	18,544249	20,6952274	18,9348801	18,7483315
14,0325947	17,6087614	16,871813	14,7082528	17,0487659	16,3865191	15,4188805
15,5346044	17,5159245	18,2418366	15,1556883	17,3712608	13,9111976	12,7758322
9,5633862	10,5415506	11,2479841	10,204247	11,348559	9,4911916	9,64470211
14,2695813	15,6679224	17,8400643	15,155853	17,4903157	14,4596507	15,8650081
16,2016411	20,8677422	19,3025351	16,3042448	19,3455496	18,7026928	21,3789142
5,84399918	6,43099199	8,13467449	6,05022649	8,15075826	6,52067217	6,61700733
14,2985625	15,5580098	16,216875	15,453563	16,8719843	16,0892691	17,4202399
10,773169	10,8739793	12,4502613	11,9015629	12,533777	10,9634921	12,4790934
13,7951441	15,5713346	17,0969361	13,853576	17,273067	14,5957937	14,340045
21,1205165	23,9638584	24,138501	21,4817816	22,3618	21,7178731	21,2808775
11,9932715	15,9406078	14,1625776	11,7182462	14,495396	11,8896579	11,9538119
10,0153891	12,5523465	12,3018491	11,0052352	11,4589259	11,049337	10,7249519
18,0864504	23,3701398	21,5491842	19,6849773	20,5639885	22,7102852	24,4397707
10,834793	12,2456651	12,6440228	11,280929	12,2615573	9,74713472	9,78552774
15,5551293	19,4085345	19,5914714	15,5352929	18,0014151	17,0336313	17,7798023
10,6718408	13,0541184	13,2831903	10,250111	12,1745474	10,9041363	11,0987898
18,5622829	20,3367785	20,4791178	18,7057647	20,0090381	21,4508786	24,0314073

0	0	0	1	1	0	0
2071049	2071049	2071049	2071050	2071050	2071051	2071051
15.02.87	15.02.82	15.02.77	15.05.08	15.09.86	15.09.86	15.09.81
NA	NA	NA	15. Apr 08	15. Apr 08	NA	NA
none	none	none	breast	breast	none	none
18,7794618	19,6724083	19,8129544	19,4961885	17,7960829	19,8831846	19,4943136
15,8133935	17,2101909	16,6042717	14,589726	16,4989887	17,2116202	19,4466276
16,3692214	15,8466192	21,872743	15,9610447	15,0885074	16,2090773	16,691853
17,4423064	18,0557778	21,2435197	18,2636793	18,215956	18,5808234	17,9806835
15,7975712	17,5140697	21,3522088	13,5886659	15,738005	16,8604171	18,5404678
14,9805039	16,4364307	23,6481107	13,7108759	16,6446415	17,6954489	18,3355027
10,6880214	10,5564745	19,7240817	8,6423927	9,22331042	10,6950896	11,1677183
14,8504433	14,9190531	19,8570029	14,2988213	15,2774083	15,135541	15,9373971
17,9859924	19,5078579	28,9505454	15,5435607	19,3961959	19,5321767	20,3773911
6,00922035	7,17407232	9,72125428	4,97493852	7,2963072	6,3662341	8,19108384
15,0700771	16,3904343	20,6903091	14,0567008	17,2882215	16,6195638	16,8113291
11,7030844	11,5393915	13,6087707	10,6940291	11,4952039	11,3283979	11,338249
15,3689101	15,4192485	21,8999452	13,5016863	14,6939448	16,309271	16,8284232
20,7555092	23,9260219	6,08013456	20,0913124	22,8145578	22,197027	23,5176867
12,2145719	13,017989	27,5465046	11,452811	12,992184	12,9445573	14,0670406
11,3266074	12,6361761	20,0144019	9,98205764	11,112655	12,5611065	13,8062049
21,4730075	20,7308033	18,6941337	19,1086997	17,2481999	20,3097806	20,0828409
11,4921981	11,7668969	18,7036006	10,2414032	11,198503	12,4506396	13,3598782
17,6273367	18,9693491	19,1598167	14,2555093	18,5407392	18,9674384	20,7162715
11,976189	13,3810288	19,1227954	9,11302284	11,9242914	12,8137662	14,7681772
17,3442446	16,885634	23,6921079	17,7949612	17,4897765	17,928408	18,1999403

0	1	1	1	1	1	1
2071051	2071052	2071052	2071053	2071053	2071053	2071053
15.09.76	15.11.98	15.09.87	15.06.03	15.06.86	15.02.82	15.01.77
NA	15. Jun 98	15. Jun 98	15may2003	15may2003	15may2003	15may2003
none	lung	lung	colon	colon	colon	colon
23,1730731	19,6054947	19,3322976	19,0269665	19,5853522	17,02923	22,2939698
18,7833818	16,5912172	16,2814829	18,121619	18,1256401	17,2815714	17,651551
17,242043	15,9922879	19,7071	14,6340996	17,3861403	16,8522342	17,6569735
18,0612575	19,7547644	19,2664642	16,5841312	18,6595301	19,5132004	20,2957235
18,1954911	16,0025074	18,7353541	17,9127301	17,9505876	16,2809991	16,7581571
18,2964546	16,7784743	16,6995221	17,9826512	18,4805636	18,767486	19,7408218
10,603402	11,9446069	10,8573399	11,5216927	12,0328779	10,8953849	11,0031026
17,3446682	14,5940578	16,898067	17,6257492	16,5512274	14,8232509	17,7477585
24,9835537	17,5501267	25,9047532	21,4080161	19,7739535	21,3000103	24,6375897
8,55457077	6,41649153	10,1843423	8,66384816	8,87572642	8,15961886	9,61944122
20,0983286	14,2305027	20,0449737	15,7845712	16,9763536	14,9053958	18,6442485
14,84504	10,5181363	12,6775136	11,4488628	12,5614022	10,3302651	13,4577638
20,0386665	14,5698193	15,5112015	17,5692437	16,4731653	15,2551982	18,1262438
25,8605562	23,0637234	24,1106781	23,6963457	22,6674349	23,0753489	27,410634
17,8442396	13,319303	15,5677871	13,9368447	13,2301546	14,8713829	18,6468333
13,4435268	12,1965821	14,1799763	13,0425826	14,0151502	12,4059337	13,4144418
26,5291189	21,4867367	32,2263325	21,0761873	19,1871434	18,0068281	26,3134049
13,0588284	11,9568818	10,5967573	12,3654843	13,1800162	11,6805363	11,7535558
23,399952	16,8361294	21,8416724	21,993732	19,4067693	19,567124	21,8624025
15,8504704	12,1688548	14,78509	13,7465795	13,8475403	13,4914859	15,4793814
18,5778235	18,0799191	23,4264748	16,2869337	19,2123057	18,7623994	19,4510305

1	1	1	1	1	1	1
2071055	2071055	2071055	2071055	2071056	2071056	2071058
15.03.01	15.09.87	15.11.85	15.03.81	15.01.99	15.06.87	15.12.95
15mar1999	15mar1999	15mar1999	15mar1999	15. Nov 98	15. Nov 98	15. Sep 95
colon	colon	colon	colon	colon	colon	breast
20,3035578	22,0067146	21,4299894	20,9084691	21,2484396	18,7460363	21,4593732
16,0115379	14,5757928	14,1155789	14,6807974	15,7858054	17,288643	16,329464
16,6648565	17,3731805	16,1015365	17,7929732	17,6266907	19,7679437	20,2844293
18,47012	17,6362018	18,0627744	19,8224999	18,4269595	30,5030709	18,6447438
15,170725	14,0839447	14,6779537	14,779601	15,9585886	19,9453426	18,7785337
16,1661359	15,940254	15,0968922	17,1308306	17,5672689	18,9471985	16,3746687
10,0862655	10,0903733	10,3395255	9,99416747	9,49956005	11,5033612	9,2761901
14,8864614	14,1391872	15,281354	14,1879708	17,5005712	18,6523824	16,5233823
16,7410574	16,2820896	15,427753	16,1192404	19,8037012	25,2991614	26,1066662
6,13050871	5,9082454	6,22640197	6,99303543	8,61800541	10,8381188	10,0254
14,6894341	15,0428243	14,5178297	14,1653485	15,2935935	19,9158364	19,1439535
11,6962962	11,2452522	11,4837092	10,5375739	11,4611713	13,9894024	13,4658835
14,9089137	13,3637545	13,946036	13,6171951	16,3252879	15,7320175	16,5019168
22,4386385	20,4533231	20,59328	22,0151724	25,2321984	24,2400886	25,7686574
13,1320146	11,2494243	11,4183847	11,9133755	13,5620369	13,0084026	15,5891354
11,3318985	9,96580195	10,2569608	10,3663951	11,7317312	14,6290637	13,7854871
19,6750592	20,0102092	20,3469152	20,2980413	22,5846423	29,5646142	35,8803619
10,9517314	10,7856639	11,0881411	10,8495813	10,8127963	12,8101091	11,2884937
16,3869743	15,3269608	15,1066636	15,7679632	17,3667864	22,1529129	22,9106702
11,0153598	10,1223269	10,0277375	11,0497673	11,5278619	15,186833	14,7337477
18,2578942	19,3123296	18,4381692	20,1236099	19,4232443	23,1029489	23,2556012

1	1	1	1	1	1	1
2071058	2071059	2071059	2071060	2071060	2071061	2071061
15.03.88	15.04.89	15.09.81	15.02.89	15.04.82	15.05.00	15.04.88
15. Sep 95	15mar1989	15mar1989	15may1988	15may1988	15mar2000	15mar2000
breast	lung	lung	colon	colon	lung	lung
20,5470829	18,7858092	17,9597955	18,390868	19,3977523	19,8034715	21,7984694
17,0792118	17,7633418	17,0440938	18,4176095	18,9435278	16,6873661	16,5830323
18,3469756	14,4338508	16,925145	18,3168205	18,7195308	19,8351136	18,4078512
19,6171885	18,2520273	18,932057	19,1599483	21,293695	18,3420851	16,7961645
17,5576509	18,1156199	15,9387023	18,2930334	19,8782477	17,6077271	15,7795625
15,5475584	20,6843589	17,7935591	17,4477202	17,5089311	18,0475918	17,0050538
9,46073315	9,84251637	11,7148635	10,3425322	11,1380946	9,2949742	9,16649219
15,5199731	13,2154583	16,6094356	16,5142528	17,4257856	17,0280658	15,453701
24,8668204	16,8162643	19,8847086	21,9823091	28,4409315	20,147609	20,4785117
9,72764597	9,09012677	7,97876443	11,4370285	12,460705	9,17794939	9,33681392
19,6057483	14,8509199	17,1842526	16,8334742	20,2695355	16,3702215	16,0991573
13,7007696	9,51742229	12,886839	12,7893919	13,3681116	12,9285852	11,6889297
16,3176465	14,530327	15,7960656	16,9548458	17,8333502	16,5578286	14,824339
25,33388	21,5060797	23,5347468	27,9635669	27,8838881	24,3431764	22,4452014
14,1552561	13,9928349	14,836687	15,5023209	17,8523216	15,0493747	13,7701963
13,5947944	13,3682082	12,7084679	14,0006125	15,2046375	12,2481951	12,3356369
24,4445928	16,8102167	22,9802998	25,1284005	28,3326088	24,1083142	22,0715208
11,0452714	12,3644936	12,8199607	11,6843152	11,8430396	11,6627208	10,028063
22,0167343	17,3473694	19,4526222	19,1819301	24,31536	20,015014	19,0302636
13,898264	11,3763273	13,4090584	12,4399141	17,1678149	13,2954994	12,835637
21,1967015	15,6058619	18,6983403	21,9601048	21,542431	21,7226003	19,430091

1	1	1	1	0	0	0
2071063	2071063	2071063	2071063	2071064	2071064	2071064
15.05.01	15.05.87	15.05.82	15.04.77	15.03.86	15.03.81	15.03.76
15. Jul 00	15. Jul 00	15. Jul 00	15. Jul 00	NA	NA	NA
colon	colon	colon	colon	none	none	none
20,0136775	20,6083997	18,6134753	20,577056	16,6175097	19,3679753	21,4046819
17,4185579	15,9087079	19,263639	18,3859767	16,3506495	17,3983889	18,5666748
17,3967943	16,8580854	17,7022409	14,2304553	15,9860796	16,0694643	17,1563696
19,7092874	18,57813	21,6818855	19,3168757	17,4019875	18,020252	18,2062309
16,9785707	16,1921844	17,1354092	17,1954532	16,2771073	16,404276	18,2473777
16,7240364	16,8227048	18,8857442	19,4141469	16,1816984	16,3427107	19,1759524
11,0559735	9,86960102	10,6194962	9,9811572	10,7405878	10,7921944	10,5827716
17,2473085	16,5383068	18,0891307	17,3575785	14,0106239	15,2484936	15,684771
19,2298384	19,5863173	20,8680249	22,3821268	19,3698069	19,7737134	27,2218704
7,49040786	7,59896623	8,31102278	9,36038557	6,53776592	6,62222984	7,99639392
16,4667016	17,0826551	17,3743246	17,4512142	15,5656081	16,4737781	16,8149414
11,644345	11,6294622	11,8469324	11,9549952	11,0540049	12,047074	11,9037585
15,2081304	15,469969	15,9794965	17,5955077	15,0373256	16,1165145	19,1113246
22,9449207	24,7918233	23,1674461	28,2738058	22,1136224	22,5393097	25,3869213
14,7380225	13,8508854	14,6621522	18,017822	12,6939041	13,0309153	17,8410691
12,2421569	11,6780363	13,0724645	12,270562	12,4266911	13,1479826	13,4143855
20,4368225	19,1133481	20,1598387	20,778109	22,5559652	22,0649426	21,6761915
12,7697502	11,7309567	13,0123547	11,941985	11,3866153	12,2151231	12,0047142
18,7339418	18,5604022	19,7176179	21,4930464	18,3661756	19,5073429	21,6005379
12,1554546	12,3917642	13,4965388	15,3501512	12,6502904	12,800271	15,7305414
19,8997483	19,2478093	19,7343688	16,0775212	16,9106597	17,0005	18,1720235

0	0	0	1	1	1	1
2071065	2071065	2071065	2071066	2071066	2071067	2071067
15.02.89	15.11.87	15.10.86	15.06.06	15.02.89	15.02.08	15.12.90
NA	NA	NA	15. Apr 06	15. Apr 06	15. Jan 04	15. Jan 04
none	none	none	lung	lung	colon	colon
20,809041	18,8310445	22,0744786	18,8683048	19,2532542	18,5952964	21,4135018
14,896063	17,2376393	16,9384329	14,4190326	14,2915415	17,1972235	16,1108363
16,1597198	15,8900248	16,6760864	20,7412961	17,2509701	16,6705396	16,3699324
17,7449325	18,8838754	17,9434295	18,2490525	17,6381918	18,2542738	19,3116906
15,490349	16,0536761	16,632208	16,2824411	14,3904423	16,295182	14,9284495
14,3432669	14,960427	17,2891113	13,1723364	14,4812445	16,087493	16,3654495
9,0077265	10,5473424	10,4559847	10,437361	8,88499423	9,14540226	9,52387325
14,4780332	16,2753935	15,9328203	15,0432258	14,6735396	14,876889	15,3472356
17,4913916	17,161049	17,7856108	20,249384	17,963203	16,71249	18,8643636
5,61123231	6,95078045	6,11130742	6,77956049	6,95334255	7,11288464	6,86816881
14,512068	15,3580877	15,2221182	16,7829111	14,2150607	15,9344658	14,9405162
11,5719028	11,7552007	12,1720167	12,0465153	10,9162682	11,4545891	10,5990788
14,7868293	15,5141892	15,3366137	14,755808	13,4680557	15,2532007	14,5938912
20,4326686	18,7847601	21,8364372	21,2529301	20,8258785	22,0134494	22,5733241
11,6624491	12,5705674	12,8961718	12,6043519	11,7165393	11,305032	13,2415874
11,0969375	11,7889608	12,1503726	11,9272619	10,0509255	11,3566315	10,8468954
20,7892051	21,3859775	19,8847532	26,6903448	21,4923055	17,9268433	19,8838008
11,2156157	12,6538075	12,3620699	9,81827647	9,0098476	11,4664739	11,2358293
15,9702789	16,8676715	17,7820059	18,6117568	15,7872962	16,8862576	17,1687975
10,4993758	11,7410155	12,2101661	11,2715598	10,2092866	11,6865674	11,947048
17,3808104	16,9845623	17,8460602	22,6086753	19,4473079	18,4150549	18,5903244

0	0	0	1	1	0	0
2071068	2071068	2071068	2071069	2071069	2071070	2071070
15.01.88	15.01.83	15.01.78	15.11.04	15.12.84	15.11.86	15.11.81
NA	NA	NA	15. Jul 03	15. Jul 03	NA	NA
none	none	none	colon	colon	none	none
20,4582224	21,9168763	21,3153364	20,3756906	18,8134626	24,9299964	19,5409012
17,3677268	19,3456081	18,0791695	14,3736226	15,6321505	16,6042717	18,2514318
16,9873349	16,8284596	15,6604077	18,4145031	19,700843	32,427962	16,7968638
17,99911	18,7319806	17,3768113	16,5919603	17,061731	23,1274557	17,7691864
18,174672	18,2995374	17,443667	15,2468123	16,7454532	22,5929213	17,6333727
17,7716978	17,9307009	19,0210306	13,407967	14,3549172	16,7729806	16,8917507
10,7013915	11,3797352	10,6552295	8,51116736	9,34256118	13,7499304	9,84056189
16,1574914	17,547045	16,6805579	14,155519	15,6006618	20,3692362	16,3878699
21,5807681	21,478129	22,0326087	21,6338046	21,1998498	27,8407352	24,2559228
7,67748645	8,18932228	8,06943266	6,55490426	8,34259072	12,3164643	7,46139804
16,7067705	17,6859366	17,5215272	17,5561734	18,019272	18,7350022	16,438735
12,0002982	12,5207072	12,1735289	12,0072386	12,2074923	14,1184141	11,9945934
16,7765003	17,6556774	18,4252213	16,3107639	15,2485301	21,9617038	18,7626154
24,584163	22,8296709	26,0081349	23,1523178	23,3212282	23,069167	24,5978839
13,4958946	13,2261845	17,3720357	14,3048989	13,7825803	25,3145223	15,5073782
13,3016341	13,9590121	13,6174139	11,0258835	12,1653314	22,9047183	13,1743337
21,1780125	23,9323286	21,9388715	29,5270585	27,1082575	23,7523276	22,7039323
12,0954769	12,873434	11,8407429	8,60640102	9,24850123	18,4013762	11,7765845
20,4073429	19,6536343	22,0062293	19,4079026	20,1261326	29,8383066	21,8426651
14,1491814	14,4835812	15,8717017	11,8736701	13,0383236	22,4823306	14,973206
17,8717372	18,3805113	16,8771476	20,3476072	21,3280746	19,4562995	17,3570088

0	1	1	1	1	1	1
2071070	2071071	2071071	2071072	2071072	2071074	2071074
15.11.76	15.05.99	15.10.88	15.03.01	15.08.88	15.01.09	15.06.89
NA	15mar1999	15mar1999	15oct1999	15oct1999	15dec2008	15dec2008
none	lung	lung	colon	colon	lung	lung
19,8756743	17,5231958	20,7893557	19,8096978	19,109837	19,5608764	19,4397434
17,7024368	15,6775806	16,2914965	16,8252256	16,4824439	14,5636598	14,827771
17,4452522	17,2169193	20,2575351	16,5812167	16,7522011	17,2795157	13,1355892
18,5499428	17,5214116	18,4601078	17,9150525	18,7663706	17,6339413	19,4608926
17,8453423	15,5622329	17,7218511	15,137552	16,30741	14,4930593	15,9919195
17,4817989	15,6271235	16,0274891	17,1219345	14,8196748	14,802867	15,1607215
9,82489486	10,0415183	10,4159789	10,0306538	10,0921726	9,82965005	9,97406505
17,4414287	14,5989769	17,5476373	15,5149036	15,8938098	14,6627149	15,3809331
22,2722583	19,7001527	27,3135916	19,2021542	19,3700708	19,3746942	20,76314
8,03227035	8,45527661	9,84033625	6,99167868	6,99417531	5,19747058	6,93703981
16,503797	16,8888781	20,8738653	15,8934688	16,9683571	16,5116864	16,7745089
12,4399073	11,7997908	13,2104293	11,9757098	12,3022006	11,063052	11,8407265
17,3067041	14,4819903	15,9622092	16,5322439	14,755556	14,3262661	14,8230517
23,9101929	23,3967504	25,7016312	23,3218899	23,3921001	21,0841538	21,7130309
18,3060204	12,9066279	15,8965833	14,7531834	12,9022399	12,296972	12,7359439
12,4072763	12,682406	13,6672694	11,8455507	11,2300342	11,0818549	11,1503758
21,3563216	22,6121243	32,3797129	19,625261	21,6817898	26,2844939	23,5474878
11,4816924	10,3309649	10,8334071	11,6175286	11,5839696	9,04673862	9,46391816
20,9355281	17,8146418	22,1606678	18,6279892	18,4990054	16,3759169	18,4682677
15,1902733	11,2036697	14,8056656	12,7422615	12,1973794	10,1179788	11,7287999
19,2004095	20,2164947	24,4866737	18,0260203	18,7163765	20,2029711	15,9033181

1	1	1	1	0	0	1
2071075	2071075	2071076	2071076	2071078	2071078	2071079
15.12.95	15.03.88	15.11.87	15.04.86	15.08.86	15.08.81	15.02.00
15. Feb 92	15. Feb 92	15oct1987	15oct1987	NA	NA	15. Jul 95
breast	breast	breast	breast	none	none	breast
21,5636072	20,9105935	18,8340373	20,7002849	21,2158052	19,6563507	20,8152654
19,2456189	15,6463566	20,7413689	16,3141151	16,1833851	17,1976575	12,6289347
19,5432167	15,9540507	17,4606578	18,6028801	16,6868976	15,3601125	16,3565932
18,3696613	19,0889643	20,9163553	20,2745293	19,2558452	18,9948105	15,8579965
19,4413508	18,6052587	20,0240851	19,2301009	17,5055582	16,8146743	12,4977585
18,5275778	15,0328759	19,443025	18,1251901	16,2861235	17,8263041	10,8735036
10,837773	10,5407445	12,9893787	10,8330711	9,60904268	9,82093107	7,89432962
17,672856	17,2913538	16,897604	17,4413675	15,7302407	15,2474797	12,9386688
23,8916179	23,1368715	21,4377139	26,7312719	20,6262966	18,8777313	15,0993603
12,6913304	9,24021165	8,05952738	10,0212611	6,81769384	6,8791851	4,5540699
18,001691	18,0070574	16,956862	20,2397065	17,551715	16,239305	13,3605131
14,3059475	13,6984449	12,6695315	13,0037707	12,8727786	11,7126453	9,70924142
18,1786504	16,0043364	17,3688781	17,0050346	16,1402637	15,5435711	11,9607098
27,2961253	24,5077141	28,8250653	24,0882742	23,1271232	22,9074138	19,447275
16,0253876	14,1488694	17,6397604	14,8568315	12,7543932	12,4592763	10,0918418
14,871599	13,1022857	14,799618	14,7307617	12,2686875	12,3357945	8,19363679
27,1925972	29,0810185	21,5406994	30,2357404	23,2808528	20,0103717	19,3094508
11,8618181	10,8853409	16,3588095	10,7653983	12,3979747	12,5738283	8,13714801
21,4662552	21,1686404	21,7798126	23,7712399	20,00993	19,1890174	13,5453492
14,5524616	13,973752	15,7638007	14,9127396	12,8703115	13,0812335	8,10570077
22,5353411	18,5083649	19,6356876	22,3994005	17,7615189	16,5866757	17,7144954

1	1	1	1	1	0	0
2071079	2071080	2071080	2071081	2071081	2071082	2071082
15.12.88	15.05.08	15.03.82	15.09.08	15.08.81	15.03.87	15.02.82
15. Jul 95	15. Aug 06	15. Aug 06	15. Aug 08	15. Aug 08	NA	NA
breast	colon	colon	breast	breast	none	none
21,3027406	19,3967442	18,3239713	19,0100301	18,8454774	20,7900846	17,7503753
16,3962486	15,701129	16,8442567	12,8312476	16,0193303	18,7173316	16,6042717
20,62329	18,8738464	19,1423323	17,0627841	16,765317	16,6341004	14,7127274
20,2431889	18,5584271	20,0641513	16,9002674	18,6433204	19,3309831	18,1942605
18,6756507	16,8043451	18,801512	13,3517356	15,8172067	16,9893512	18,3646314
15,0239989	14,7241912	18,0637203	12,8321322	14,9871716	18,36501	19,4660777
10,7367113	9,73684349	10,757203	8,39108863	9,41216421	10,9662053	10,8394385
16,4480669	15,0432486	17,2356685	14,2002976	15,5361565	15,5897247	14,9325088
23,1444366	21,6629303	24,5624407	15,9943345	19,0967071	19,3463658	19,2470236
9,29912272	6,86596798	11,4555961	4,54755336	7,47963322	8,22888245	8,48868119
18,2698879	17,3822067	19,6451593	13,9015237	16,66231	16,1155014	16,3267498
13,2125656	12,2021047	13,9070486	10,4297969	11,4650861	11,6925674	10,8930949
15,6081657	15,545422	16,6639986	12,8994943	13,854619	15,9021271	16,2696686
24,0135938	23,124885	23,7627901	18,9304387	21,8202954	24,3016344	23,2034442
14,4056168	13,8408246	16,8622085	11,0702834	12,2550249	13,5154294	14,6605559
13,3623709	12,5453367	14,0723917	9,12298541	11,4675657	13,2799511	13,8044403
26,5322094	27,6464684	29,2928071	20,8093196	20,1806875	19,9913513	19,4502572
11,6573624	9,6668942	11,958729	9,07859406	11,3858359	12,362557	12,7922936
21,2995755	19,5356761	22,7971023	14,1154351	18,178174	20,4261383	21,0029469
14,1513845	11,8992582	16,2070455	8,51202704	11,5855695	14,4128478	14,7649304
23,4154338	23,4686739	21,8965369	18,8200512	18,8171939	17,6667215	15,8485326

1	1	0	0	0	1	1
2071083	2071083	2071085	2071085	2071085	2071086	2071086
15.03.06	15.03.89	15.04.80	15.10.78	15.07.77	15.05.00	15.06.91
15mar2006	15mar2006	NA	NA	NA	15. Nov 99	15. Nov 99
lung	lung	none	none	none	breast	breast
18,1845602	19,8989466	19,184615	18,1835783	19,8129544	21,5297659	19,5712083
14,8562323	15,7948386	16,4717631	16,1455445	16,6042717	15,1322347	13,8453463
15,9272367	19,0144612	15,9130464	15,7658582	17,4818408	19,3371642	18,3349518
17,5313198	19,4175846	20,5485709	17,6060046	19,0250005	17,4821969	19,1913285
15,2985417	17,4858719	16,6202177	15,537125	16,7608111	16,6460488	15,6728491
14,4194487	15,0513373	14,5536701	15,1418728	16,7729806	13,6700866	13,7819843
9,46457688	10,6773938	10,5698963	10,1067606	10,5533267	9,77550018	9,98519133
14,8674712	17,2702618	15,4061587	13,1057216	15,9627607	15,6155525	15,844877
16,6527709	23,2963569	17,3701433	18,745154	20,5236514	20,4547891	21,2584992
6,2572425	9,11205721	5,88570112	5,96648029	8,02432	7,57477599	7,28656127
16,2971812	19,4973831	15,8126395	15,7186882	16,7806806	18,5807643	18,2991771
11,8358666	13,0258442	11,3184558	10,8558375	12,0438308	12,4387635	12,3954372
13,7816459	15,9906062	14,564399	14,8818572	15,7857395	15,8508053	15,0793884
20,9619967	23,9648982	20,6405927	19,5228033	23,069167	21,7143338	21,7591155
11,8572978	14,2176623	12,196005	12,7725912	14,0622145	12,8644962	12,7546384
10,5944529	12,8360668	11,6449395	11,4546884	12,4886794	12,8913281	11,2317557
21,3002091	30,1033771	23,7470559	23,1532035	22,3386964	27,4283719	28,6404834
10,7556193	10,9447467	11,4676066	11,6459812	11,7947995	9,18064129	10,0436568
16,3974884	21,5137501	17,2269124	17,5803862	19,1598167	18,1789539	18,3826072
10,0028406	13,9830753	11,6234407	12,0319177	12,9938054	11,6825501	11,243831
18,1750347	22,3949488	17,223115	17,2880638	19,4562995	21,1013549	21,5863451

1	1	0	0	0	1	1
2071087	2071087	2071088	2071088	2071088	2071089	2071089
15.05.95	15.03.73	15.05.77	15.07.76	15.10.74	15.10.03	15.05.86
15. Jan 93	15. Jan 93	NA	NA	NA	15. Jul 03	15. Jul 03
colon	colon	none	none	none	breast	breast
18,1509083	16,9947934	16,4653658	17,9744524	19,8129544	19,0994331	24,1190173
17,0034565	15,9528668	15,81656	15,6419175	16,6042717	13,9923663	16,4693428
18,8058742	21,7496397	16,9676963	17,1799979	17,4818408	18,9916474	18,7836186
18,3182916	21,2412089	18,4043008	17,6841847	19,0250005	18,1612473	19,2475892
16,4480211	19,5450108	15,5584223	15,4651761	16,7608111	15,2134397	17,8574306
18,5889616	18,0585552	14,1936373	15,5587303	16,7729806	14,2938348	19,2072901
10,2989417	11,0598353	8,49009094	10,1360229	10,5533267	9,30803395	10,2417567
16,2646333	22,852898	14,7992013	14,1045951	15,9627607	15,3418262	17,0484102
21,3436025	25,9191409	15,7118526	18,3594355	20,5236514	19,6412433	21,3322504
9,47865067	10,0767206	8,17046755	6,42093683	8,02432	6,01390556	9,9642033
16,6623485	20,4073546	14,2561336	15,3348179	16,7806806	16,5033185	16,2057641
11,5957959	12,3821427	8,70722346	10,8165883	12,0438308	11,2598542	12,1231354
15,5304037	15,7421244	8,67650742	14,3118373	15,7857395	14,7467171	16,2679296
23,3547227	25,113633	16,4593645	18,5922262	23,069167	21,762061	26,6722349
15,1102148	16,8485603	7,8715694	12,1538915	14,0622145	12,07746	15,26871
12,299521	13,2314188	9,14925352	11,2528882	12,4886794	11,3677461	13,1591673
21,6386781	22,3386964	17,6463324	23,9011571	22,3386964	25,8645584	20,9656052
10,5641427	11,8545751	11,6428081	11,0101931	11,7947995	9,17737583	11,995196
18,7711383	22,1559608	16,8259267	18,0762575	19,1598167	17,2169823	19,8713361
12,9452092	14,071391	10,8729227	11,785447	12,9938054	10,7330983	13,8282494
20,4994429	22,1418196	19,4667112	18,0214708	19,4562995	22,1556104	20,70487

0	0	0	1	1	1	1
2071090	2071090	2071090	2071093	2071093	2071094	2071094
15.12.86	15.01.82	15.01.77	15.02.89	15.11.81	15.08.99	15.12.86
NA	NA	NA	15. Sep 86	15. Sep 86	15. Apr 98	15. Apr 98
none	none	none	breast	breast	colon	colon
20,8435244	18,6710748	22,0504452	20,4756513	19,6014643	18,4535944	18,5967798
18,8285348	16,6954682	19,0905767	17,4033511	16,0571527	16,2012719	16,4456421
16,3232271	16,6702204	18,0513374	18,2410418	18,2175146	17,3336948	17,2606878
20,1933818	19,5877194	19,27964	18,7520342	17,8658208	20,5101277	18,6150319
17,855867	15,9065639	19,3384387	19,7846375	17,2826423	15,5296531	15,9478832
17,3638558	17,4126629	17,6095821	18,7842239	16,2645415	15,7642566	16,5895121
11,5500696	10,3384036	10,4180066	10,1455884	10,0980003	11,3496126	9,90730781
16,5692455	14,7970708	17,5883239	16,8310205	15,969554	15,1950293	15,1691657
20,70714	18,5982379	23,981489	22,2267258	25,5800577	19,8599322	18,713561
8,90605445	7,4223998	9,20663774	10,5810018	9,798349	6,12694763	6,68600247
18,2793496	16,2649653	16,5549564	16,0258152	20,1091575	16,1472384	16,1758765
12,8745772	11,6015809	12,6970852	11,8558647	13,064796	11,6511593	11,6617044
16,6851848	15,4340709	16,7668934	19,1664643	16,6685157	15,1334553	14,7749378
22,8645046	23,9352919	25,7241625	26,3192277	23,9217648	23,0639081	21,9692341
13,2664474	13,6027618	19,0015387	16,0975204	14,2393833	12,449613	13,3690471
13,2233212	11,5518757	12,5647861	14,6048632	13,9182997	11,7363517	11,7938851
20,5744985	19,1678624	24,9007087	23,3009286	27,8412274	20,5235771	19,264791
13,8880147	12,1516448	12,6326035	12,0507616	10,5935664	12,4559782	11,9073191
20,6141923	18,6883477	22,4722061	20,9981029	22,7402023	16,3519072	17,9725327
14,3351984	13,1095032	15,6379156	13,7832825	14,3654029	11,3109967	12,3071605
18,5885228	18,0883067	20,696543	21,425761	20,6008311	19,7515842	19,4996688

1	1	1	1	1	1	1
2071094	2071094	2071095	2071095	2071096	2071096	2071097
15.12.81	15.12.76	15.05.07	15.05.88	15.08.06	15.05.89	15.09.09
15. Apr 98	15. Apr 98	15. Apr 07	15. Apr 07	15. Jul 06	15. Jul 06	15mar2009
colon	colon	breast	breast	lung	lung	lung
19,3921407	21,4996344	20,4203278	21,581501	18,380845	17,0873185	19,4546837
16,4451054	19,9448897	13,5892553	14,4099097	14,7988071	16,1075313	15,8450651
15,5883203	18,4832343	17,9114369	19,0037075	19,5711911	20,3282189	13,1609
19,9455724	20,3014463	18,0006347	20,6066007	20,0068028	19,050941	15,9988928
16,7891611	19,4331072	14,7535196	17,3498963	16,948599	16,4608972	13,3076438
17,9950072	20,3466835	13,6128417	13,8519772	14,3042726	15,215796	12,6699397
11,1547043	11,30241	9,32661561	10,5705074	9,96720717	9,21960232	8,94913993
15,9496569	19,0136415	14,2125852	16,1738394	15,1068254	16,0087807	13,9085306
20,5398145	26,5965202	19,229018	22,0413627	19,5374727	22,404589	15,282595
8,01317715	10,654944	6,64471462	8,66481563	8,20648828	9,07782406	4,57024931
17,6189279	17,4796135	16,1532225	16,9480869	17,1618271	17,5964574	12,6616778
12,7566077	11,9374806	11,1602624	12,8002431	11,8799683	12,3382118	9,53556756
16,0909192	19,6428097	14,5341935	15,2646564	16,0414404	14,6279034	13,8204029
24,2926784	25,9231105	22,3019402	22,8626831	23,6334831	22,6787661	21,1767307
13,3321165	21,4974346	12,2849694	13,5799386	13,7498054	12,2250502	10,5443367
12,6474068	14,1548362	11,0137736	11,7385519	11,9750266	11,992237	10,6006212
23,0917702	23,0671931	24,5760237	26,7147856	27,2079564	27,7243506	19,3927419
12,7100026	13,5223753	9,30032191	10,6204699	10,6641481	10,9588656	9,0675387
19,3647228	23,2592438	16,8202561	18,7527569	19,234066	20,1577414	13,0441911
13,7118304	17,7770924	10,182168	12,3532304	12,5841475	12,6914167	7,95962095
17,598907	20,7072856	20,6548771	22,2761209	21,6699303	22,7554661	15,2887325

1	1	1	1	1	1	1
2071097	2071099	2071099	2071100	2071100	2071101	2071101
15.03.88	15.03.95	15.04.86	15.04.99	15.10.89	15.07.91	15.01.87
15mar2009	15. Nov 94	15. Nov 94	15. Jan 99	15. Jan 99	15. Jun 91	15. Jun 91
lung	lung	lung	breast	breast	lung	lung
18,9112585	17,7747412	20,200301	21,659512	22,8487574	18,5249982	19,3470755
15,8445908	18,8830421	16,4017001	13,7438223	13,68839	18,4346666	17,419237
16,0713686	18,0454768	18,263926	19,6410919	19,0978405	18,737394	18,2210849
17,6744823	21,3140415	17,9546157	18,9258362	17,7613185	22,8614185	20,6715324
16,0392886	19,240861	18,6817249	15,3216719	15,2929634	19,1199727	16,1116309
17,7061104	19,71557	16,4059633	12,2543079	13,1989151	16,5944896	16,9289141
10,491601	12,5613548	10,3953191	9,67581725	8,83029845	10,8019104	11,0963614
15,4384785	19,9853274	16,200189	15,1103007	15,3506176	15,1827357	16,642195
19,5026504	23,5177266	25,7783623	18,4362172	17,5737528	24,488852	18,7932655
7,65357519	10,1489794	10,4160011	6,89115295	7,62151915	9,41774416	7,28161567
16,2247975	17,5750801	20,4876856	16,1717808	14,8950755	17,7955933	17,200416
11,4171806	11,9349239	13,8091855	11,9396511	11,7068015	12,1474512	12,5817155
15,6419143	18,0517326	17,1731413	14,2225429	13,6593381	16,5040279	15,5734401
22,7099547	27,4427669	26,3374168	22,0163439	21,9082558	27,6143228	22,7948571
13,1711924	16,9488515	15,4694959	12,1632518	12,8771912	14,8640802	14,3386102
11,8843677	14,4826531	13,9619154	11,110241	10,1370825	14,5722253	13,0833453
20,3085183	22,2459666	23,7679428	24,2015313	19,9419467	26,8708287	18,1225135
10,8656703	13,7210777	11,449024	10,112809	10,4109236	12,7708029	13,5136872
18,9197587	23,3669796	23,5452149	17,042937	16,4964166	21,356487	19,2816641
12,7806588	16,4552088	15,1411014	10,8142587	10,8794949	13,5474768	12,4734488
17,7309084	20,7981792	20,8677664	22,0548443	22,601929	22,2547663	20,2691604

1	1	1	1	1	1	0
2071102	2071102	2071103	2071103	2071103	2071103	2071105
15.01.96	15.09.85	15.03.08	15.08.86	15.08.81	15.08.76	15.01.87
15. Jan 96	15. Jan 96	15mar2007	15mar2007	15mar2007	15mar2007	NA
colon	colon	colon	colon	colon	colon	none
19,5492501	22,1248262	18,9311386	19,4443965	19,0134939	21,0801189	18,9931288
17,3432643	16,2194994	15,0318132	17,815479	15,8410326	18,3316835	16,2604891
19,1890957	20,0443821	15,5871556	17,4808989	17,156941	17,761092	15,7656811
19,4636183	21,5034761	17,5573676	19,3715919	18,7052307	18,1672234	19,3099122
18,0496898	18,5323577	13,6328828	16,8701122	15,1148058	16,9197147	16,1696001
17,3704087	17,7705413	15,1411108	18,0092599	16,9876279	19,4005878	15,5839273
11,4021832	11,6854089	9,64207619	10,5461401	10,501949	10,3263222	10,6754815
16,2680562	18,0754835	14,5971692	15,4934948	14,8852007	16,6907395	14,9068505
23,4483927	25,3125879	15,3790188	20,1322057	18,5585009	22,8162453	17,7183045
10,1608315	10,2425249	5,43600193	8,24029758	6,66766166	8,51947112	5,92788302
18,3657715	18,2462405	14,0342581	16,9006516	16,0156738	17,4097073	15,609883
13,6803536	13,9811956	10,7250515	11,4363476	10,9956337	12,6977174	11,239418
16,7959007	16,2370396	14,2898568	16,0292461	15,040686	17,1911819	15,172734
25,1978077	24,8181898	21,7235573	22,8047039	23,8525927	25,8678521	19,976494
14,918746	14,9028252	11,0383503	14,0465661	12,59193	17,9585776	12,4109423
13,4186324	14,0693038	10,5688687	12,6361454	11,4056697	12,1041126	12,087391
26,6551981	29,2725864	18,5158452	19,7852529	19,870293	20,8276699	20,5767134
11,3076338	11,5254175	10,8047548	12,4215827	11,7096059	11,8474912	12,2322818
21,8397572	20,7500521	13,6874946	19,3598023	17,4592381	22,2344366	17,8761856
13,7829089	13,8875055	8,8313748	13,6930396	12,0219503	15,3210834	12,1207671
21,6211242	22,7024249	17,8056983	19,6175508	18,9015437	19,7879677	17,1848865

0	0	1	1	0	0	0
2071105	2071105	2071106	2071106	2071107	2071107	2071107
15.01.82	15.01.77	15.06.92	15.08.76	15.09.86	15.09.81	15.09.76
NA	NA	15. Apr 91	15. Apr 91	NA	NA	NA
none	none	breast	breast	none	none	none
17,6187911	19,1924915	19,1275549	19,7185285	21,865311	19,7521052	20,2578782
17,2598254	19,7906692	18,2315339	13,9710775	17,1231064	16,957142	16,8865488
16,4127119	16,8289568	16,7836473	17,5025492	17,2994756	16,022785	17,5442542
19,1461894	18,7250905	19,0858804	18,5971455	21,0679534	23,484437	21,3813183
16,9973751	19,6097688	16,2459201	15,1537785	16,4637168	16,2501924	17,2948145
17,5094908	18,9951018	15,10481	12,7709998	16,7528444	16,5276085	17,9264344
11,1645721	11,631415	11,9921335	9,7834107	11,1535115	10,6571459	10,6816719
14,5778807	19,8893319	14,8484035	14,2461618	15,8917305	15,6453968	18,685072
18,4736466	23,2219039	16,7820089	15,9839613	19,2151265	19,3375694	22,4664877
7,07784148	10,1952054	6,9151852	6,74631816	8,05577998	7,2805294	8,7653083
16,0257986	19,7333169	15,2357316	16,0187269	16,3472109	17,085716	17,5484152
10,9207393	12,6588711	11,7610133	11,168244	12,0095128	13,0020622	12,7702124
15,4453731	19,7100786	14,8969602	12,6625637	15,3039222	16,1485843	17,3486276
21,4097501	28,3032734	22,5740586	20,8402334	23,7523197	22,689382	26,9739302
12,9410848	18,199613	12,4494977	10,4186637	14,0255223	13,0414359	17,9337689
13,0931729	14,830074	13,1073956	10,1770711	12,203668	12,3120369	12,6849203
20,4819974	23,1033645	21,5077819	21,4671173	19,8527672	21,3255011	23,3088425
13,4824853	14,3125536	13,2636251	11,2712431	12,912091	12,8290903	12,5571101
18,3338824	22,9503856	15,977189	16,4227187	18,6371349	18,2209034	21,5689242
13,3837749	17,3676251	10,3910227	10,1697004	13,1629856	12,147897	15,2198501
17,2648246	17,868467	18,3610964	19,6692374	19,4249544	18,0052336	19,4994533

0	0	0	0	0	0	0
2071108	2071108	2071108	2071109	2071109	2071110	2071110
15.10.91	15.03.87	15.02.82	15.12.81	15.10.79	15.10.87	15.11.85
NA	NA	NA	NA	NA	NA	NA
none	none	none	none	none	none	none
18,3778863	19,7372752	17,8138021	20,531945	20,7335349	20,5941535	20,6458841
16,2330102	17,1395366	17,6256917	15,0813206	14,5562559	14,3196404	16,1073348
15,1881622	13,6352848	16,9064346	16,8078797	16,4793876	16,1910762	17,6983606
16,9636169	17,8932166	18,5832915	18,3352455	17,9916216	17,8094282	19,8204908
16,2487622	17,3657784	17,0927792	15,2299645	14,5878696	13,8703881	16,0774525
15,6038218	17,6114729	17,9319813	15,6678568	15,135772	15,0346481	17,3485961
9,29172383	10,5221834	10,664982	9,67718579	9,37110947	9,7192663	10,1525422
14,7593526	15,4141506	14,3928105	14,670819	14,1076715	13,9972146	14,691349
18,5662345	19,331127	20,4771532	19,3405509	16,2300448	15,282391	17,6457137
6,73758182	7,58638604	7,82255224	6,72267528	6,54279881	5,72065467	7,83358826
16,9438657	17,0987877	16,4407573	15,1602657	13,8652081	14,5086115	14,2065541
12,037092	10,7799995	11,2652096	11,3744853	10,7972732	11,0085582	11,0735188
15,0681441	15,3694709	15,3458528	14,5287911	13,898992	13,345515	14,8955399
21,8029117	22,5313207	22,7474191	21,7758429	20,0053685	19,8425415	22,8053486
10,928317	12,6932148	10,5906891	12,3187839	12,0958429	11,0773152	13,3313785
11,873086	13,0209761	13,3978905	10,4701085	9,58336774	9,94411794	11,4123885
21,6901783	20,1103639	21,4649723	22,0469723	19,0843431	18,5925604	18,4776901
10,67773	11,9053602	12,6471805	10,6679064	10,1994436	10,6065645	11,045807
17,9816118	18,8919204	18,8767137	16,5341459	15,6098418	14,4150327	17,2157619
11,6179716	12,9198236	12,8147832	10,6608574	10,3410724	9,62641551	12,0009402
16,3157155	14,5904402	18,2538281	18,6076256	18,1364268	18,1842899	19,6534998

0	1	1	0	0	0	0
2071110	2071112	2071112	2071113	2071113	2071113	2071115
15.03.81	15.05.08	15.05.81	15.11.86	15.09.81	15.10.76	15.06.86
NA	15. Apr 08	15. Apr 08	NA	NA	NA	NA
none	lung	lung	none	none	none	none
20,0611058	18,0245315	21,0861197	17,3190419	18,4587448	20,705054	18,3030443
15,4596825	16,0026671	17,3117036	16,2636124	17,3969502	18,8186155	18,1723071
13,0809209	16,9474545	18,2077919	15,7633734	16,0607041	16,5122554	18,2466371
17,4708337	21,4702085	20,946445	17,4575358	18,7442526	19,7141318	20,9500264
14,9129383	14,5094611	19,399359	16,2979264	16,6987829	18,8995124	18,2175606
15,5847165	15,6700759	17,2203524	15,5475882	16,7448398	19,5250315	19,2710821
9,74720681	10,8051919	11,116077	9,41884647	10,1591939	10,8478112	11,336222
14,1540823	13,6037683	16,731303	15,4396422	15,8400904	17,5074625	18,0690811
15,8533184	17,6327608	26,2765919	19,9334657	19,1975302	23,0538133	21,4889087
6,25472351	5,3282829	10,4769744	6,692294	7,46918261	8,57539313	7,74247061
13,9622632	13,5285756	21,3555835	18,4984752	17,2653812	18,0699144	18,9037193
10,5300022	9,8508236	12,8887545	12,9351718	11,7444699	12,467277	12,077788
13,5899914	14,5663581	16,9199229	15,1636146	15,7760141	23,9086935	15,1650864
21,9521096	21,6937278	26,3689472	21,1756182	26,0454743	24,1377681	21,7710219
11,4125159	13,1903856	13,8304717	12,9627273	13,7743295	17,7409358	14,0775502
10,4666839	11,3297831	14,1086465	11,965813	12,4201028	13,6901344	12,2853935
17,8974496	19,4589533	22,3386964	24,7906293	22,1775148	22,462408	21,0666764
10,5956807	11,6678359	12,0272363	11,626355	12,2167517	12,6851059	13,5879871
15,6855867	15,1268352	21,9823829	19,1747979	19,1529817	22,8469205	19,6675887
10,3750935	10,4989533	14,9362088	12,2241055	13,5799051	16,1687143	13,6096001
15,0335801	19,3613017	21,0723126	16,9139652	17,2465935	17,6577186	20,7202592

0	0	0	0	0	1	1
2071115	2071115	2071116	2071116	2071116	2071117	2071117
15.02.82	15.02.77	15.05.87	15.05.82	15.05.77	15.07.91	15.04.86
NA	NA	NA	NA	NA	15. Apr 91	15. Apr 91
none	none	none	none	none	colon	colon
17,0854505	22,1336948	19,1528605	18,6210889	20,5982311	20,7177055	23,4780355
18,4059944	18,4735569	14,7159967	16,3688055	17,2897399	15,7148198	15,8087597
17,2649036	17,141565	16,6848284	16,8949174	16,8390312	18,0102558	18,0859462
19,7490351	18,8478904	17,2344777	17,6292858	18,1910194	18,8608485	19,0525985
18,2464064	18,49905	13,9433756	15,496934	16,7526738	19,0783774	17,8596288
18,2985507	19,5087118	14,816639	16,1879683	17,7511889	16,7881431	16,2121009
10,8149987	10,6804731	9,02029897	9,45116762	9,81194772	10,9134109	10,6149018
15,4489061	17,0862584	14,2014969	14,1611215	16,585285	16,2781345	15,8339092
21,1756757	23,7678544	17,352583	18,1649431	22,6419771	23,4577301	22,3613032
7,94863668	9,14796774	6,13427015	7,17570695	8,98006238	9,80132395	9,57949082
16,9924937	16,8558139	15,3077815	16,1844779	16,753059	19,466025	18,3328144
11,1543163	12,4873638	11,1676514	11,3715896	11,9100367	13,3382547	12,7179466
15,7920632	18,3718228	13,8939837	14,1792095	16,9819824	16,2209893	15,9801799
23,0637629	25,3406045	20,7611237	23,4273239	24,9376943	25,5094767	24,0030093
13,9598612	17,3817369	11,8562841	12,5065022	17,8506103	14,3703312	14,6953314
13,4685482	12,6064052	10,1686356	11,5128717	12,3855323	13,616586	13,4114235
21,4824098	25,0137187	19,3655871	18,9416728	23,7597186	27,9090739	23,7114226
13,3342989	12,4165096	9,92833644	10,8166542	10,861469	10,6458889	11,2003064
20,997034	20,2412947	15,7108252	17,8157366	20,6643409	21,1928692	20,1040102
14,8448014	15,200747	10,961098	11,9777113	14,9953048	13,7015497	13,8104816
18,4483372	19,1163117	18,2484819	18,4268612	18,5761459	21,3709393	20,7864322

1	1	1	1	1	1
2071119	2071119	2071119	2071119	2071120	2071120
15.10.06	15.09.86	15.09.81	15.09.76	15.05.02	15.01.88
15. Sep 06	15. Sep 06	15. Sep 06	15. Sep 06	15. Jan 00	15. Jan 00
colon	colon	colon	colon	breast	breast
18,7022213	19,3330014	18,9865091	21,0068974	18,0580536	19,6075017
14,5971815	19,4759415	17,2629752	17,4029453	14,7285303	17,0295007
16,9331987	16,5802697	17,1089985	16,9323246	17,9465801	21,2593523
18,9257462	20,194336	19,3217683	20,1348326	17,1087829	20,6819083
14,2577555	16,3887411	16,7696734	16,97796	14,5044615	19,6039197
14,7306763	19,6826475	18,1960632	20,3364269	14,6805671	17,3379384
10,0444097	11,3674382	11,0863721	11,2869402	8,9440688	11,9809534
15,1897759	16,6507334	16,3212167	17,6761146	13,403002	19,1305391
16,7837084	21,1463989	19,8598508	23,46316	16,8760356	24,7321162
5,46934747	8,48486524	8,29221596	10,1631657	5,7715495	10,6688624
15,2017523	17,728236	17,5244841	18,0720141	13,6276154	20,5933501
11,3396941	10,9305854	12,3258776	12,6080021	9,94804828	14,5985965
14,0142592	15,0666228	16,1023909	16,8076326	13,8243247	17,2655203
19,7650379	24,1104543	25,9526683	25,0591012	21,3846487	25,3845823
11,6611274	14,7869747	13,8198086	17,5768567	12,6149008	15,3991204
9,97386907	13,2219271	12,7167517	12,7415162	10,4508967	15,2151933
22,0452931	17,9799967	19,6447164	21,9152585	19,1838128	24,3367271
10,7461201	13,2043574	12,7195365	12,8151451	9,75297263	12,7616407
15,5433017	20,0518329	18,754783	21,5906959	16,1383313	23,2927887
9,72741989	13,5640542	13,5666605	15,0966067	10,3215213	15,527023
18,9061626	18,7073021	18,5967354	18,5862977	20,0040546	23,6646624

Cacostat	1	1	0	1	0	1
PID	2071087	2071040	2071005	2071008	2071088	2071025
Date.of.blood.draw	15.03.73	15.08.73	15.09.74	15.09.74	15.10.74	15.11.74
Date.of.diagnosis	15. Jan 93	15. Feb 94	NA	15. Apr 08	NA	15. Feb 04
type	colon	colon	none	colon	none	colon
hsa-let-7b-3p	16,9947934	19,893384	20,3250157	20,3560948	19,8129544	18,2766391
hsa-let-7d-3p	15,9528668	18,7129397	12,8433007	16,6451078	16,6042717	13,7484986
miR-100-5p	21,7496397	22,1890895	17,8979492	18,7882499	17,4818408	17,1325816
miR-10a-5p	21,2412089	21,1369026	18,4125826	18,4237256	19,0250005	18,5128995
miR-140-5p	19,5450108	20,7382777	11,7770113	16,2640426	16,7608111	11,9591162
miR-149-3p	18,0585552	19,4044527	12,3346617	17,3146112	16,7729806	12,027318
miR-150-5p	11,0598353	11,9097815	8,35216293	9,56562685	10,5533267	9,08856808
miR-155-5p	22,852898	18,6273612	13,2720837	16,8781736	15,9627607	14,0907183
miR-186-5p	25,9191409	31,0537216	13,5661912	21,115437	20,5236514	13,1446356
miR-223-3p	10,0767206	14,4856739	3,76989357	9,20205254	8,02432	3,66279312
miR-29c-5p	20,4073546	21,9081148	12,8760777	17,1820633	16,7806806	12,9335581
miR-30a-5p	12,3821427	15,588824	9,3686817	13,4398792	12,0438308	9,30148114
miR-328-5p	15,7421244	21,1223507	12,1377205	16,1093837	15,7857395	11,5400103
miR-423-3p	25,113633	23,069167	16,4654581	23,5773734	23,069167	17,0299781
miR-484	16,8485603	14,0622145	9,55554052	16,2306211	14,0622145	9,05492729
miR-5006-5p	13,2314188	16,947566	8,48954087	11,57322	12,4886794	8,86344092
miR-5196-5p	22,3386964	35,0018077	17,3902341	23,5170164	22,3386964	17,508687
miR-575	11,8545751	13,308088	8,86645927	11,0237882	11,7947995	8,82727104
miR-630	22,1559608	27,4037801	9,5067869	19,9661855	19,1598167	10,4112047
miR-6821-5p	14,071391	20,4961876	5,97547496	13,3067434	12,9938054	6,20709491
miR-99a-5p	22,1418196	24,526533	19,6710051	20,7570293	19,4562995	19,5537767

0	0	1	0	0	0	1
2071064	2071031	2071025	2071088	2071032	2071035	2071103
15.03.76	15.04.76	15.07.76	15.07.76	15.08.76	15.08.76	15.08.76
NA	NA	15. Feb 04	NA	NA	NA	15mar2007
none	none	colon	none	none	none	colon
21,4046819	19,8129544	17,7963755	17,9744524	18,3192371	20,2669666	21,0801189
18,5666748	16,6042717	16,1812625	15,6419175	19,5106279	22,3892975	18,3316835
17,1563696	17,4818408	17,4660241	17,1799979	17,0200828	16,2283737	17,761092
18,2062309	19,0250005	22,7406235	17,6841847	18,658325	20,9092602	18,1672234
18,2473777	16,7608111	16,6745318	15,4651761	17,7772824	22,6230151	16,9197147
19,1759524	16,7729806	17,8554096	15,5587303	19,3284526	23,3137745	19,4005878
10,5827716	10,5533267	11,879681	10,1360229	11,5536006	10,9268316	10,3263222
15,684771	15,9627607	15,1805165	14,1045951	16,4869676	16,8463262	16,6907395
27,2218704	20,5236514	18,8572647	18,3594355	21,7292168	25,3069952	22,8162453
7,99639392	8,02432	7,46143047	6,42093683	9,01560557	7,3328395	8,51947112
16,8149414	16,7806806	16,1500626	15,3348179	18,1676934	18,6106321	17,4097073
11,9037585	12,0438308	11,0660073	10,8165883	12,6868451	11,6961737	12,6977174
19,1113246	15,7857395	13,7541908	14,3118373	18,8722025	17,7528368	17,1911819
25,3869213	23,069167	21,9773843	18,5922262	24,0207544	25,8130806	25,8678521
17,8410691	14,0622145	12,2838094	12,1538915	17,33842	20,8354487	17,9585776
13,4143855	12,4886794	11,3874544	11,2528882	13,6367058	13,989184	12,1041126
21,6761915	22,3386964	21,4998495	23,9011571	22,5511651	23,4956527	20,8276699
12,0047142	11,7947995	14,1793516	11,0101931	13,3317014	14,834551	11,8474912
21,6005379	19,1598167	18,2883561	18,0762575	21,3847016	24,9594583	22,2344366
15,7305414	12,9938054	11,8614433	11,785447	16,1954372	16,7600496	15,3210834
18,1720235	19,4562995	20,180572	18,0214708	18,274755	17,273556	19,7879677

1	0	0	0	1	0	0
2071106	2071003	2071051	2071107	2071119	2071015	2071018
15.08.76	15.09.76	15.09.76	15.09.76	15.09.76	15.10.76	15.10.76
15. Apr 91	NA	NA	NA	15. Sep 06	NA	NA
breast	none	none	none	colon	none	none
19,7185285	19,1002534	23,1730731	20,2578782	21,0068974	20,0701112	22,144407
13,9710775	17,4656085	18,7833818	16,8865488	17,4029453	17,9888302	17,9177443
17,5025492	17,8261364	17,242043	17,5442542	16,9323246	17,5421505	16,9426994
18,5971455	20,7814475	18,0612575	21,3813183	20,1348326	18,9271622	20,3407227
15,1537785	18,7556638	18,1954911	17,2948145	16,97796	16,9589378	17,590023
12,7709998	20,18059	18,2964546	17,9264344	20,3364269	19,1322516	22,3534206
9,7834107	8,93432007	10,603402	10,6816719	11,2869402	11,5088585	9,76583055
14,2461618	17,7255721	17,3446682	18,685072	17,6761146	17,5677932	17,8859601
15,9839613	25,8220229	24,9835537	22,4664877	23,46316	24,6916599	25,5962558
6,74631816	11,1336726	8,55457077	8,7653083	10,1631657	9,21713324	10,9810935
16,0187269	16,7168859	20,0983286	17,5484152	18,0720141	18,1128772	17,5725249
11,168244	11,3656126	14,84504	12,7702124	12,6080021	12,7384823	13,1000705
12,6625637	17,586246	20,0386665	17,3486276	16,8076326	18,2232613	17,079909
20,8402334	27,006524	25,8605562	26,9739302	25,0591012	25,1946386	25,2470829
10,4186637	22,1730595	17,8442396	17,9337689	17,5768567	18,7003196	18,8496693
10,1770711	12,2089025	13,4435268	12,6849203	12,7415162	13,2595146	13,6117363
21,4671173	22,7945565	26,5291189	23,3088425	21,9152585	22,6103314	22,724215
11,2712431	10,9854102	13,0588284	12,5571101	12,8151451	12,6423432	12,2199548
16,4227187	23,518394	23,399952	21,5689242	21,5906959	20,6426331	23,7436278
10,1697004	16,4484175	15,8504704	15,2198501	15,0966067	15,5916349	16,2876023
19,6692374	20,486941	18,5778235	19,4994533	18,5862977	19,6561856	19,1889978

0	0	1	1	1	0	0
2071113	2071070	2071094	2071019	2071053	2071090	2071105
15.10.76	15.11.76	15.12.76	15.01.77	15.01.77	15.01.77	15.01.77
NA	NA	15. Apr 98	15. Nov 97	15may2003	NA	NA
none	none	colon	colon	colon	none	none
20,705054	19,8756743	21,4996344	22,2700091	22,2939698	22,0504452	19,1924915
18,8186155	17,7024368	19,9448897	17,3223621	17,651551	19,0905767	19,7906692
16,5122554	17,4452522	18,4832343	18,3912614	17,6569735	18,0513374	16,8289568
19,7141318	18,5499428	20,3014463	18,477067	20,2957235	19,27964	18,7250905
18,8995124	17,8453423	19,4331072	17,784943	16,7581571	19,3384387	19,6097688
19,5250315	17,4817989	20,3466835	18,7643311	19,7408218	17,6095821	18,9951018
10,8478112	9,82489486	11,30241	10,6746544	11,0031026	10,4180066	11,631415
17,5074625	17,4414287	19,0136415	17,3202981	17,7477585	17,5883239	19,8893319
23,0538133	22,2722583	26,5965202	25,9535477	24,6375897	23,981489	23,2219039
8,57539313	8,03227035	10,654944	9,58303915	9,61944122	9,20663774	10,1952054
18,0699144	16,503797	17,4796135	16,5923688	18,6442485	16,5549564	19,7333169
12,467277	12,4399073	11,9374806	13,5247179	13,4577638	12,6970852	12,6588711
23,9086935	17,3067041	19,6428097	19,3465311	18,1262438	16,7668934	19,7100786
24,1377681	23,9101929	25,9231105	27,7732778	27,410634	25,7241625	28,3032734
17,7409358	18,3060204	21,4974346	18,0454583	18,6468333	19,0015387	18,199613
13,6901344	12,4072763	14,1548362	13,4174102	13,4144418	12,5647861	14,830074
22,462408	21,3563216	23,0671931	20,9525094	26,3134049	24,9007087	23,1033645
12,6851059	11,4816924	13,5223753	12,4381852	11,7535558	12,6326035	14,3125536
22,8469205	20,9355281	23,2592438	21,6114532	21,8624025	22,4722061	22,9503856
16,1687143	15,1902733	17,7770924	15,3775611	15,4793814	15,6379156	17,3676251
17,6577186	19,2004095	20,7072856	20,0550483	19,4510305	20,696543	17,868467

0	0	1	0	0	0	0
2071049	2071115	2071063	2071088	2071116	2071085	2071012
15.02.77	15.02.77	15.04.77	15.05.77	15.05.77	15.07.77	15.01.78
NA	NA	15. Jul 00	NA	NA	NA	NA
none	none	colon	none	none	none	none
19,8129544	22,1336948	20,577056	16,4653658	20,5982311	19,8129544	19,680161
16,6042717	18,4735569	18,3859767	15,81656	17,2897399	16,6042717	19,6372108
21,872743	17,141565	14,2304553	16,9676963	16,8390312	17,4818408	17,4732805
21,2435197	18,8478904	19,3168757	18,4043008	18,1910194	19,0250005	21,839281
21,3522088	18,49905	17,1954532	15,5584223	16,7526738	16,7608111	20,2291327
23,6481107	19,5087118	19,4141469	14,1936373	17,7511889	16,7729806	21,869504
19,7240817	10,6804731	9,9811572	8,49009094	9,81194772	10,5533267	11,2811114
19,8570029	17,0862584	17,3575785	14,7992013	16,585285	15,9627607	20,0157989
28,9505454	23,7678544	22,3821268	15,7118526	22,6419771	20,5236514	30,1810914
9,72125428	9,14796774	9,36038557	8,17046755	8,98006238	8,02432	9,97984024
20,6903091	16,8558139	17,4512142	14,2561336	16,753059	16,7806806	19,5277711
13,6087707	12,4873638	11,9549952	8,70722346	11,9100367	12,0438308	13,399331
21,8999452	18,3718228	17,5955077	8,67650742	16,9819824	15,7857395	18,0936194
6,08013456	25,3406045	28,2738058	16,4593645	24,9376943	23,069167	27,0544805
27,5465046	17,3817369	18,017822	7,8715694	17,8506103	14,0622145	21,6944902
20,0144019	12,6064052	12,270562	9,14925352	12,3855323	12,4886794	13,8794926
18,6941337	25,0137187	20,778109	17,6463324	23,7597186	22,3386964	25,4788868
18,7036006	12,4165096	11,941985	11,6428081	10,861469	11,7947995	14,2698409
19,1598167	20,2412947	21,4930464	16,8259267	20,6643409	19,1598167	22,8673629
19,1227954	15,200747	15,3501512	10,8729227	14,9953048	12,9938054	17,0090329
23,6921079	19,1163117	16,0775212	19,4667112	18,5761459	19,4562995	19,3274171

0	1	0	0	1	0	1
2071068	2071044	2071085	2071109	2071047	2071085	2071055
15.01.78	15.02.78	15.10.78	15.10.79	15.12.79	15.04.80	15.03.81
NA	15. Feb 03	NA	NA	15. Sep 99	NA	15mar1999
none	colon	none	none	colon	none	colon
21,3153364	19,4318788	18,1835783	20,7335349	22,437302	19,184615	20,9084691
18,0791695	19,1645911	16,1455445	14,5562559	15,3499611	16,4717631	14,6807974
15,6604077	17,9651061	15,7658582	16,4793876	17,862331	15,9130464	17,7929732
17,3768113	19,7206959	17,6060046	17,9916216	20,6952274	20,5485709	19,8224999
17,443667	17,4422375	15,537125	14,5878696	17,0487659	16,6202177	14,779601
19,0210306	20,2126327	15,1418728	15,135772	17,3712608	14,5536701	17,1308306
10,6552295	10,8940196	10,1067606	9,37110947	11,348559	10,5698963	9,99416747
16,6805579	19,701692	13,1057216	14,1076715	17,4903157	15,4061587	14,1879708
22,0326087	22,7697562	18,745154	16,2300448	19,3455496	17,3701433	16,1192404
8,06943266	8,92679781	5,96648029	6,54279881	8,15075826	5,88570112	6,99303543
17,5215272	17,5922246	15,7186882	13,8652081	16,8719843	15,8126395	14,1653485
12,1735289	11,9874819	10,8558375	10,7972732	12,533777	11,3184558	10,5375739
18,4252213	17,7203285	14,8818572	13,898992	17,273067	14,564399	13,6171951
26,0081349	24,8497553	19,5228033	20,0053685	22,3618	20,6405927	22,0151724
17,3720357	19,0429326	12,7725912	12,0958429	14,495396	12,196005	11,9133755
13,6174139	12,7566901	11,4546884	9,58336774	11,4589259	11,6449395	10,3663951
21,9388715	23,1682304	23,1532035	19,0843431	20,5639885	23,7470559	20,2980413
11,8407429	13,0125616	11,6459812	10,1994436	12,2615573	11,4676066	10,8495813
22,0062293	20,9662944	17,5803862	15,6098418	18,0014151	17,2269124	15,7679632
15,8717017	15,6521713	12,0319177	10,3410724	12,1745474	11,6234407	11,0497673
16,8771476	19,7203754	17,2880638	18,1364268	20,0090381	17,223115	20,1236099

0	0	0	1	1	0	0
2071064	2071110	2071031	2071112	2071042	2071035	2071078
15.03.81	15.03.81	15.05.81	15.05.81	15.06.81	15.08.81	15.08.81
NA	NA	NA	15. Apr 08	15. Jun 91	NA	NA
none	none	none	lung	lung	none	none
19,3679753	20,0611058	19,4870556	21,0861197	20,3233777	20,2335209	19,6563507
17,3983889	15,4596825	17,5177991	17,3117036	17,0249595	19,1692207	17,1976575
16,0694643	13,0809209	16,0348017	18,2077919	21,4220492	16,1121695	15,3601125
18,020252	17,4708337	17,913261	20,946445	19,635959	20,3094171	18,9948105
16,404276	14,9129383	16,6961187	19,399359	18,8759724	20,2593672	16,8146743
16,3427107	15,5847165	17,5481969	17,2203524	17,983865	20,7844583	17,8263041
10,7921944	9,74720681	10,3032522	11,116077	11,0252341	11,5455921	9,82093107
15,2484936	14,1540823	15,0216638	16,731303	18,6576225	16,4306149	15,2474797
19,7737134	15,8533184	19,088408	26,2765919	24,5207018	25,2031148	18,8777313
6,62222984	6,25472351	7,18573293	10,4769744	10,8786632	8,02438272	6,8791851
16,4737781	13,9622632	16,7641454	21,3555835	20,0034588	17,3113467	16,239305
12,047074	10,5300022	11,6779467	12,8887545	13,3875998	11,8901374	11,7126453
16,1165145	13,5899914	16,4172004	16,9199229	16,6603914	20,9609016	15,5435711
22,5393097	21,9521096	22,9747622	26,3689472	24,6974781	25,3590582	22,9074138
13,0309153	11,4125159	12,6671712	13,8304717	15,3607149	17,0126212	12,4592763
13,1479826	10,4666839	12,8516142	14,1086465	13,8694181	14,5616183	12,3357945
22,0649426	17,8974496	20,8509692	22,3386964	25,1783214	21,7594577	20,0103717
12,2151231	10,5956807	11,9831344	12,0272363	11,4741938	14,2386311	12,5738283
19,5073429	15,6855867	19,1591233	21,9823829	22,2875944	21,6118525	19,1890174
12,800271	10,3750935	13,4035119	14,9362088	15,1685254	16,4945824	13,0812335
17,0005	15,0335801	16,9309027	21,0723126	22,7121137	17,5927313	16,5866757

1	1	0	0	1	0	0
2071081	2071103	2071003	2071018	2071020	2071032	2071051
15.08.81	15.08.81	15.09.81	15.09.81	15.09.81	15.09.81	15.09.81
15. Aug 08	15mar2007	NA	NA	15. Aug 95	NA	NA
breast	colon	none	none	lung	none	none
18,8454774	19,0134939	18,2455571	19,4907866	20,5218004	20,4623303	19,4943136
16,0193303	15,8410326	16,9550176	16,4870609	16,1994512	17,2764349	19,4466276
16,765317	17,156941	16,7449023	16,9461898	17,6350205	16,0729773	16,691853
18,6433204	18,7052307	17,4605876	18,6191433	17,5835004	17,9724346	17,9806835
15,8172067	15,1148058	15,8096868	16,6448285	18,0567351	17,8987813	18,5404678
14,9871716	16,9876279	17,8244361	17,0373661	15,8235711	18,9819365	18,3355027
9,41216421	10,501949	9,08301888	10,7949355	9,34362467	10,5178575	11,1677183
15,5361565	14,8852007	16,2529413	16,6827416	15,2840891	15,5357311	15,9373971
19,0967071	18,5585009	21,4067499	19,220589	21,7481367	20,9822835	20,3773911
7,47963322	6,66766166	8,72223421	7,47344611	9,55019884	7,30267022	8,19108384
16,66231	16,0156738	16,2710152	16,5102673	17,0177318	15,8526783	16,8113291
11,4650861	10,9956337	10,4392292	11,5766208	11,6485612	11,4333371	11,338249
13,854619	15,040686	15,4902168	16,7742148	15,9293582	16,2185866	16,8284232
21,8202954	23,8525927	22,7526319	21,6661954	23,7375996	22,9311387	23,5176867
12,2550249	12,59193	12,7011877	12,6520178	13,5078436	12,9669921	14,0670406
11,4675657	11,4056697	11,8662853	12,5424941	12,26639	12,9726792	13,8062049
20,1806875	19,870293	18,9281043	22,6811591	25,1294065	23,4539937	20,0828409
11,3858359	11,7096059	10,0908134	12,0824688	10,2648461	12,0830853	13,3598782
18,178174	17,4592381	19,049671	19,4347701	19,5053286	19,055013	20,7162715
11,5855695	12,0219503	12,6403358	13,1227664	13,2376842	12,8325382	14,7681772
18,8171939	18,9015437	19,0747261	17,9453595	20,9134351	17,4213067	18,1999403

1	0	0	1	0	1	0
2071059	2071107	2071113	2071119	2071015	2071047	2071070
15.09.81	15.09.81	15.09.81	15.09.81	15.10.81	15.11.81	15.11.81
15mar1989	NA	NA	15. Sep 06	NA	15. Sep 99	NA
lung	none	none	colon	none	colon	none
17,9597955	19,7521052	18,4587448	18,9865091	19,2099998	21,6560874	19,5409012
17,0440938	16,957142	17,3969502	17,2629752	17,0747121	14,7164785	18,2514318
16,925145	16,022785	16,0607041	17,1089985	17,7281711	16,9276002	16,7968638
18,932057	23,484437	18,7442526	19,3217683	19,2898536	18,544249	17,7691864
15,9387023	16,2501924	16,6987829	16,7696734	15,9985133	14,7082528	17,6333727
17,7935591	16,5276085	16,7448398	18,1960632	18,1014787	15,1556883	16,8917507
11,7148635	10,6571459	10,1591939	11,0863721	10,0487157	10,204247	9,84056189
16,6094356	15,6453968	15,8400904	16,3212167	16,0749347	15,155853	16,3878699
19,8847086	19,3375694	19,1975302	19,8598508	20,0928028	16,3042448	24,2559228
7,97876443	7,2805294	7,46918261	8,29221596	7,62062684	6,05022649	7,46139804
17,1842526	17,085716	17,2653812	17,5244841	15,2146677	15,453563	16,438735
12,886839	13,0020622	11,7444699	12,3258776	10,655819	11,9015629	11,9945934
15,7960656	16,1485843	15,7760141	16,1023909	14,7518961	13,853576	18,7626154
23,5347468	22,689382	26,0454743	25,9526683	22,5158694	21,4817816	24,5978839
14,836687	13,0414359	13,7743295	13,8198086	14,2085221	11,7182462	15,5073782
12,7084679	12,3120369	12,4201028	12,7167517	11,690933	11,0052352	13,1743337
22,9802998	21,3255011	22,1775148	19,6447164	18,691255	19,6849773	22,7039323
12,8199607	12,8290903	12,2167517	12,7195365	11,7496069	11,280929	11,7765845
19,4526222	18,2209034	19,1529817	18,754783	18,2737731	15,5352929	21,8426651
13,4090584	12,147897	13,5799051	13,5666605	12,733882	10,250111	14,973206
18,6983403	18,0052336	17,2465935	18,5967354	19,7704471	18,7057647	17,3570088

1	0	1	0	1	1	0
2071093	2071005	2071094	2071109	2071019	2071025	2071090
15.11.81	15.12.81	15.12.81	15.12.81	15.01.82	15.01.82	15.01.82
15. Sep 86	NA	15. Apr 98	NA	15. Nov 97	15. Feb 04	NA
breast	none	colon	none	colon	colon	none
19,6014643	19,2526363	19,3921407	20,531945	19,7781546	22,8298634	18,6710748
16,0571527	16,8582057	16,4451054	15,0813206	17,8096031	15,3526468	16,6954682
18,2175146	17,4629967	15,5883203	16,8078797	17,5693147	16,8698573	16,6702204
17,8658208	19,5274948	19,9455724	18,3352455	18,9632895	18,6376335	19,5877194
17,2826423	15,9349179	16,7891611	15,2299645	16,1608788	14,860282	15,9065639
16,2645415	16,9405376	17,9950072	15,6678568	19,4396691	16,1082023	17,4126629
10,0980003	10,2913127	11,1547043	9,67718579	10,4973502	10,6868542	10,3384036
15,969554	15,5857799	15,9496569	14,670819	15,79788	14,9552488	14,7970708
25,5800577	20,8078269	20,5398145	19,3405509	20,77328	17,4618742	18,5982379
9,798349	7,58422442	8,01317715	6,72267528	8,38827161	7,01023185	7,4223998
20,1091575	17,1807704	17,6189279	15,1602657	17,1749722	14,6307009	16,2649653
13,064796	12,4109836	12,7566077	11,3744853	12,1001479	11,4032872	11,6015809
16,6685157	14,5585038	16,0909192	14,5287911	16,6224856	14,6588332	15,4340709
23,9217648	21,6903564	24,2926784	21,7758429	25,8205577	22,8969654	23,9352919
14,2393833	11,9898653	13,3321165	12,3187839	15,530725	12,7749073	13,6027618
13,9182997	12,034093	12,6474068	10,4701085	13,0117798	10,8671369	11,5518757
27,8412274	20,491991	23,0917702	22,0469723	21,2307019	18,8584949	19,1678624
10,5935664	12,387453	12,7100026	10,6679064	12,4911661	11,7308258	12,1516448
22,7402023	19,8251221	19,3647228	16,5341459	20,3316683	16,2862569	18,6883477
14,3654029	12,5475894	13,7118304	10,6608574	14,3926456	11,3739214	13,1095032
20,6008311	19,2864782	17,598907	18,6076256	20,0056367	19,1018284	18,0883067

0	0	1	0	0	0	1
2071105	2071049	2071053	2071082	2071108	2071115	2071080
15.01.82	15.02.82	15.02.82	15.02.82	15.02.82	15.02.82	15.03.82
NA	NA	15may2003	NA	NA	NA	15. Aug 06
none	none	colon	none	none	none	colon
17,6187911	19,6724083	17,02923	17,7503753	17,8138021	17,0854505	18,3239713
17,2598254	17,2101909	17,2815714	16,6042717	17,6256917	18,4059944	16,8442567
16,4127119	15,8466192	16,8522342	14,7127274	16,9064346	17,2649036	19,1423323
19,1461894	18,0557778	19,5132004	18,1942605	18,5832915	19,7490351	20,0641513
16,9973751	17,5140697	16,2809991	18,3646314	17,0927792	18,2464064	18,801512
17,5094908	16,4364307	18,767486	19,4660777	17,9319813	18,2985507	18,0637203
11,1645721	10,5564745	10,8953849	10,8394385	10,664982	10,8149987	10,757203
14,5778807	14,9190531	14,8232509	14,9325088	14,3928105	15,4489061	17,2356685
18,4736466	19,5078579	21,3000103	19,2470236	20,4771532	21,1756757	24,5624407
7,07784148	7,17407232	8,15961886	8,48868119	7,82255224	7,94863668	11,4555961
16,0257986	16,3904343	14,9053958	16,3267498	16,4407573	16,9924937	19,6451593
10,9207393	11,5393915	10,3302651	10,8930949	11,2652096	11,1543163	13,9070486
15,4453731	15,4192485	15,2551982	16,2696686	15,3458528	15,7920632	16,6639986
21,4097501	23,9260219	23,0753489	23,2034442	22,7474191	23,0637629	23,7627901
12,9410848	13,017989	14,8713829	14,6605559	10,5906891	13,9598612	16,8622085
13,0931729	12,6361761	12,4059337	13,8044403	13,3978905	13,4685482	14,0723917
20,4819974	20,7308033	18,0068281	19,4502572	21,4649723	21,4824098	29,2928071
13,4824853	11,7668969	11,6805363	12,7922936	12,6471805	13,3342989	11,958729
18,3338824	18,9693491	19,567124	21,0029469	18,8767137	20,997034	22,7971023
13,3837749	13,3810288	13,4914859	14,7649304	12,8147832	14,8448014	16,2070455
17,2648246	16,885634	18,7623994	15,8485326	18,2538281	18,4483372	21,8965369

1	0	1	0	0	0	1
2071060	2071041	2071063	2071116	2071012	2071068	2071044
15.04.82	15.05.82	15.05.82	15.05.82	15.01.83	15.01.83	15.02.83
15may1988	NA	15. Jul 00	NA	NA	NA	15. Feb 03
colon	none	colon	none	none	none	colon
19,3977523	18,3247572	18,6134753	18,6210889	18,8218756	21,9168763	18,9508272
18,9435278	16,8689062	19,263639	16,3688055	17,7182982	19,3456081	15,295499
18,7195308	16,3654556	17,7022409	16,8949174	17,0120719	16,8284596	17,2097081
21,293695	19,0036294	21,6818855	17,6292858	21,505131	18,7319806	19,606377
19,8782477	15,3227249	17,1354092	15,496934	16,4909506	18,2995374	14,7837965
17,5089311	15,4045323	18,8857442	16,1879683	17,0844486	17,9307009	15,2667032
11,1380946	10,8413226	10,6194962	9,45116762	11,189749	11,3797352	10,1525711
17,4257856	15,9684182	18,0891307	14,1611215	15,7325233	17,547045	14,3604455
28,4409315	18,8062052	20,8680249	18,1649431	18,5775047	21,478129	16,6513142
12,460705	7,03166462	8,31102278	7,17570695	8,09691696	8,18932228	6,48333327
20,2695355	17,5659353	17,3743246	16,1844779	16,6805838	17,6859366	15,5257742
13,3681116	12,5544722	11,8469324	11,3715896	11,3548962	12,5207072	10,9919278
17,8333502	15,74035	15,9794965	14,1792095	15,1312769	17,6556774	13,985354
27,8838881	20,0909031	23,1674461	23,4273239	22,3653301	22,8296709	21,463452
17,8523216	12,6667551	14,6621522	12,5065022	13,9278056	13,2261845	12,422434
15,2046375	12,4560234	13,0724645	11,5128717	12,1643276	13,9590121	10,5797487
28,3326088	20,4652941	20,1598387	18,9416728	18,6338942	23,9323286	17,8218963
11,8430396	12,6251869	13,0123547	10,8166542	13,4991857	12,873434	11,6340508
24,31536	18,363403	19,7176179	17,8157366	17,917633	19,6536343	15,9385271
17,1678149	12,2255827	13,4965388	11,9777113	12,3114214	14,4835812	11,0693468
21,542431	17,147713	19,7343688	18,4268612	19,0136371	18,3805113	19,0836809

1	1	0	1	0	1	1
2071069	2071102	2071034	2071055	2071110	2071021	2071010
15.12.84	15.09.85	15.10.85	15.11.85	15.11.85	15.01.86	15.02.86
15. Jul 03	15. Jan 96	NA	15mar1999	NA	15mar2001	15. Feb 00
colon	colon	none	colon	none	lung	breast
18,8134626	22,1248262	20,768392	21,4299894	20,6458841	20,4218786	20,2539631
15,6321505	16,2194994	15,3419844	14,1155789	16,1073348	17,8869041	16,8818083
19,700843	20,0443821	15,7821413	16,1015365	17,6983606	17,7963785	17,1307038
17,061731	21,5034761	19,2042138	18,0627744	19,8204908	18,9391969	18,8506857
16,7454532	18,5323577	15,9888985	14,6779537	16,0774525	17,7244632	15,0703419
14,3549172	17,7705413	16,309769	15,0968922	17,3485961	19,0539311	17,586844
9,34256118	11,6854089	10,2015154	10,3395255	10,1525422	11,4573584	11,2658425
15,6006618	18,0754835	14,3104066	15,281354	14,691349	17,5160955	16,0591069
21,1998498	25,3125879	17,7386788	15,427753	17,6457137	20,6872079	18,5579985
8,34259072	10,2425249	5,70398339	6,22640197	7,83358826	9,50019677	7,57194117
18,019272	18,2462405	14,4450471	14,5178297	14,2065541	16,5622583	16,2212382
12,2074923	13,9811956	10,8100993	11,4837092	11,0735188	12,434633	12,06588
15,2485301	16,2370396	14,9241238	13,946036	14,8955399	17,5060497	15,1421775
23,3212282	24,8181898	20,6516327	20,59328	22,8053486	25,8067088	23,518237
13,7825803	14,9028252	11,5077157	11,4183847	13,3313785	16,1327541	14,0110226
12,1653314	14,0693038	11,4767985	10,2569608	11,4123885	13,7574334	11,9799828
27,1082575	29,2725864	20,4172897	20,3469152	18,4776901	22,6123035	19,4614638
9,24850123	11,5254175	11,6401064	11,0881411	11,045807	12,3313148	12,3071576
20,1261326	20,7500521	16,682519	15,1066636	17,2157619	21,3706619	18,0855632
13,0383236	13,8875055	11,391754	10,0277375	12,0009402	14,5368101	12,9630558
21,3280746	22,7024249	17,2676028	18,4381692	19,6534998	19,7033745	19,0493303

1	0	0	1	1	1	0
2071047	2071064	2071035	2071076	2071099	2071117	2071031
15.02.86	15.03.86	15.04.86	15.04.86	15.04.86	15.04.86	15.05.86
15. Sep 99	NA	NA	15oct1987	15. Nov 94	15. Apr 91	NA
colon	none	none	breast	lung	colon	none
21,8305883	16,6175097	18,4839344	20,7002849	20,200301	23,4780355	19,8626378
16,5674233	16,3506495	21,9183504	16,3141151	16,4017001	15,8087597	20,6438839
18,4815483	15,9860796	16,059386	18,6028801	18,263926	18,0859462	16,7143105
19,5915322	17,4019875	21,8023439	20,2745293	17,9546157	19,0525985	21,04839
16,871813	16,2771073	24,6037285	19,2301009	18,6817249	17,8596288	18,1425443
18,2418366	16,1816984	21,1826003	18,1251901	16,4059633	16,2121009	21,7877437
11,2479841	10,7405878	12,6722897	10,8330711	10,3953191	10,6149018	11,9165536
17,8400643	14,0106239	16,6847508	17,4413675	16,200189	15,8339092	16,6253173
19,3025351	19,3698069	26,7330578	26,7312719	25,7783623	22,3613032	21,069877
8,13467449	6,53776592	9,46303259	10,0212611	10,4160011	9,57949082	9,07861082
16,216875	15,5656081	19,2132851	20,2397065	20,4876856	18,3328144	17,3228212
12,4502613	11,0540049	11,7222099	13,0037707	13,8091855	12,7179466	11,8512054
17,0969361	15,0373256	17,6706075	17,0050346	17,1731413	15,9801799	17,5556054
24,138501	22,1136224	25,4076254	24,0882742	26,3374168	24,0030093	23,2828063
14,1625776	12,6939041	15,750625	14,8568315	15,4694959	14,6953314	14,583745
12,3018491	12,4266911	15,9014333	14,7307617	13,9619154	13,4114235	14,6787801
21,5491842	22,5559652	23,3147867	30,2357404	23,7679428	23,7114226	19,0883038
12,6440228	11,3866153	16,0549902	10,7653983	11,449024	11,2003064	13,9372666
19,5914714	18,3661756	26,4006573	23,7712399	23,5452149	20,1040102	21,7215322
13,2831903	12,6502904	16,9429229	14,9127396	15,1411014	13,8104816	16,0930572
20,4791178	16,9106597	17,3699013	22,3994005	20,8677664	20,7864322	17,9150925

1	1	0	0	1	0	1
2071089	2071053	2071115	2071032	2071045	2071078	2071103
15.05.86	15.06.86	15.06.86	15.08.86	15.08.86	15.08.86	15.08.86
15. Jul 03	15may2003	NA	NA	15dec2000	NA	15mar2007
breast	colon	none	none	lung	none	colon
24,1190173	19,5853522	18,3030443	19,0693227	18,2468775	21,2158052	19,4443965
16,4693428	18,1256401	18,1723071	18,5044119	16,8077742	16,1833851	17,815479
18,7836186	17,3861403	18,2466371	16,4753325	18,2856962	16,6868976	17,4808989
19,2475892	18,6595301	20,9500264	18,7786836	18,6564923	19,2558452	19,3715919
17,8574306	17,9505876	18,2175606	17,3861633	17,2969527	17,5055582	16,8701122
19,2072901	18,4805636	19,2710821	18,9051366	18,5641294	16,2861235	18,0092599
10,2417567	12,0328779	11,336222	11,3706084	10,407088	9,60904268	10,5461401
17,0484102	16,5512274	18,0690811	15,3812239	17,8069681	15,7302407	15,4934948
21,3322504	19,7739535	21,4889087	18,7864982	23,5922072	20,6262966	20,1322057
9,9642033	8,87572642	7,74247061	7,64808297	10,337887	6,81769384	8,24029758
16,2057641	16,9763536	18,9037193	16,5437118	17,5588431	17,551715	16,9006516
12,1231354	12,5614022	12,077788	12,1587409	12,60103	12,8727786	11,4363476
16,2679296	16,4731653	15,1650864	15,8513928	15,9253367	16,1402637	16,0292461
26,6722349	22,6674349	21,7710219	22,985508	24,2664255	23,1271232	22,8047039
15,26871	13,2301546	14,0775502	13,4919972	16,0713992	12,7543932	14,0465661
13,1591673	14,0151502	12,2853935	12,7852637	13,4374985	12,2686875	12,6361454
20,9656052	19,1871434	21,0666764	19,7965462	19,2527881	23,2808528	19,7852529
11,995196	13,1800162	13,5879871	12,9480859	12,0598494	12,3979747	12,4215827
19,8713361	19,4067693	19,6675887	19,926397	22,1429614	20,00993	19,3598023
13,8282494	13,8475403	13,6096001	13,5102784	15,0202783	12,8703115	13,6930396
20,70487	19,2123057	20,7202592	17,4148254	20,8552489	17,7615189	19,6175508

1	1	0	0	1	0	0
2071013	2071050	2071051	2071107	2071119	2071015	2071018
15.09.86	15.09.86	15.09.86	15.09.86	15.09.86	15.10.86	15.10.86
15oct1998	15. Apr 08	NA	NA	15. Sep 06	NA	NA
breast	breast	none	none	colon	none	none
19,6581171	17,7960829	19,8831846	21,865311	19,3330014	19,443162	18,5112782
16,5018215	16,4989887	17,2116202	17,1231064	19,4759415	15,4057764	16,3912152
18,0398441	15,0885074	16,2090773	17,2994756	16,5802697	16,6190759	15,649209
23,9427101	18,215956	18,5808234	21,0679534	20,194336	17,554808	17,1352964
17,0926567	15,738005	16,8604171	16,4637168	16,3887411	15,1435299	15,8091943
17,0331851	16,6446415	17,6954489	16,7528444	19,6826475	15,9041169	15,9223333
10,5277538	9,22331042	10,6950896	11,1535115	11,3674382	9,81033026	9,85459877
16,7197315	15,2774083	15,135541	15,8917305	16,6507334	15,2157605	14,2187178
19,80241	19,3961959	19,5321767	19,2151265	21,1463989	18,9780763	17,721914
7,29239194	7,2963072	6,3662341	8,05577998	8,48486524	6,31873432	6,38876098
16,7125093	17,2882215	16,6195638	16,3472109	17,728236	15,5680095	14,9423927
11,8832371	11,4952039	11,3283979	12,0095128	10,9305854	10,9903075	11,2291216
15,4955393	14,6939448	16,309271	15,3039222	15,0666228	14,8035822	15,7849527
22,0630728	22,8145578	22,197027	23,7523197	24,1104543	21,5077884	21,5433643
13,5169768	12,992184	12,9445573	14,0255223	14,7869747	12,7683255	13,1274028
11,5922029	11,112655	12,5611065	12,203668	13,2219271	10,6441871	11,7426581
22,455244	17,2481999	20,3097806	19,8527672	17,9799967	19,4646682	21,5019812
12,4154162	11,198503	12,4506396	12,912091	13,2043574	10,9318866	11,2693501
19,3061248	18,5407392	18,9674384	18,6371349	20,0518329	17,0714029	17,2881952
12,7289862	11,9242914	12,8137662	13,1629856	13,5640542	11,1709996	12,1363777
20,3803723	17,4897765	17,928408	19,4249544	18,7073021	18,8070764	16,8290397

0	0	0	1	0	0	1
2071065	2071070	2071113	2071019	2071034	2071090	2071094
15.10.86	15.11.86	15.11.86	15.12.86	15.12.86	15.12.86	15.12.86
NA	NA	NA	15. Nov 97	NA	NA	15. Apr 98
none	none	none	colon	none	none	colon
22,0744786	24,9299964	17,3190419	18,6981185	23,9311143	20,8435244	18,5967798
16,9384329	16,6042717	16,2636124	17,9739053	15,9677937	18,8285348	16,4456421
16,6760864	32,427962	15,7633734	17,9830462	16,7805165	16,3232271	17,2606878
17,9434295	23,1274557	17,4575358	20,4182823	17,9796687	20,1933818	18,6150319
16,632208	22,5929213	16,2979264	17,3852053	16,4437927	17,855867	15,9478832
17,2891113	16,7729806	15,5475882	18,3803133	16,3016605	17,3638558	16,5895121
10,4559847	13,7499304	9,41884647	10,7354952	11,1170433	11,5500696	9,90730781
15,9328203	20,3692362	15,4396422	17,9067935	16,3681908	16,5692455	15,1691657
17,7856108	27,8407352	19,9334657	21,3549959	17,9966082	20,70714	18,713561
6,11130742	12,3164643	6,692294	8,50255482	6,9576504	8,90605445	6,68600247
15,2221182	18,7350022	18,4984752	16,5582319	16,4593099	18,2793496	16,1758765
12,1720167	14,1184141	12,9351718	11,6833591	12,3249616	12,8745772	11,6617044
15,3366137	21,9617038	15,1636146	15,8261338	15,7344215	16,6851848	14,7749378
21,8364372	23,069167	21,1756182	22,972578	20,1194727	22,8645046	21,9692341
12,8961718	25,3145223	12,9627273	14,4652716	12,6254532	13,2664474	13,3690471
12,1503726	22,9047183	11,965813	12,3150206	12,282721	13,2233212	11,7938851
19,8847532	23,7523276	24,7906293	20,1349378	23,3938778	20,5744985	19,264791
12,3620699	18,4013762	11,626355	11,9594526	12,1730485	13,8880147	11,9073191
17,7820059	29,8383066	19,1747979	19,2405771	18,1855761	20,6141923	17,9725327
12,2101661	22,4823306	12,2241055	13,7832361	12,4831513	14,3351984	12,3071605
17,8460602	19,4562995	16,9139652	19,9845674	18,1998136	18,5885228	19,4996688

1	1	0	0	0	0	0
2071017	2071101	2071105	2071049	2071082	2071108	2071041
15.01.87	15.01.87	15.01.87	15.02.87	15.03.87	15.03.87	15.05.87
15. Sep 02	15. Jun 91	NA	NA	NA	NA	NA
colon	lung	none	none	none	none	none
20,3527037	19,3470755	18,9931288	18,7794618	20,7900846	19,7372752	19,0958707
17,1119656	17,419237	16,2604891	15,8133935	18,7173316	17,1395366	17,1659449
17,8904843	18,2210849	15,7656811	16,3692214	16,6341004	13,6352848	16,430344
20,6765007	20,6715324	19,3099122	17,4423064	19,3309831	17,8932166	18,0422184
17,3498428	16,1116309	16,1696001	15,7975712	16,9893512	17,3657784	16,0942721
19,4693364	16,9289141	15,5839273	14,9805039	18,36501	17,6114729	15,4197667
10,286761	11,0963614	10,6754815	10,6880214	10,9662053	10,5221834	10,8310555
17,2734709	16,642195	14,9068505	14,8504433	15,5897247	15,4141506	15,0440269
21,6780906	18,7932655	17,7183045	17,9859924	19,3463658	19,331127	18,7415345
8,17455426	7,28161567	5,92788302	6,00922035	8,22888245	7,58638604	6,80249044
16,3191473	17,200416	15,609883	15,0700771	16,1155014	17,0987877	14,8907901
11,9996658	12,5817155	11,239418	11,7030844	11,6925674	10,7799995	10,5859616
15,9324012	15,5734401	15,172734	15,3689101	15,9021271	15,3694709	15,8554812
22,7748998	22,7948571	19,976494	20,7555092	24,3016344	22,5313207	21,0409227
14,8550927	14,3386102	12,4109423	12,2145719	13,5154294	12,6932148	13,2653754
11,9842089	13,0833453	12,087391	11,3266074	13,2799511	13,0209761	12,3214454
21,418473	18,1225135	20,5767134	21,4730075	19,9913513	20,1103639	18,5307293
13,2597732	13,5136872	12,2322818	11,4921981	12,362557	11,9053602	11,8236481
19,7008145	19,2816641	17,8761856	17,6273367	20,4261383	18,8919204	18,2737971
13,8342443	12,4734488	12,1207671	11,976189	14,4128478	12,9198236	12,7636148
20,4599412	20,2691604	17,1848865	17,3442446	17,6667215	14,5904402	17,398816

1	0	1	1	1	0	0
2071063	2071116	2071056	2071052	2071055	2071110	2071065
15.05.87	15.05.87	15.06.87	15.09.87	15.09.87	15.10.87	15.11.87
15. Jul 00	NA	15. Nov 98	15. Jun 98	15mar1999	NA	NA
colon	none	colon	lung	colon	none	none
20,6083997	19,1528605	18,7460363	19,3322976	22,0067146	20,5941535	18,8310445
15,9087079	14,7159967	17,288643	16,2814829	14,5757928	14,3196404	17,2376393
16,8580854	16,6848284	19,7679437	19,7071	17,3731805	16,1910762	15,8900248
18,57813	17,2344777	30,5030709	19,2664642	17,6362018	17,8094282	18,8838754
16,1921844	13,9433756	19,9453426	18,7353541	14,0839447	13,8703881	16,0536761
16,8227048	14,816639	18,9471985	16,6995221	15,940254	15,0346481	14,960427
9,86960102	9,02029897	11,5033612	10,8573399	10,0903733	9,7192663	10,5473424
16,5383068	14,2014969	18,6523824	16,898067	14,1391872	13,9972146	16,2753935
19,5863173	17,352583	25,2991614	25,9047532	16,2820896	15,282391	17,161049
7,59896623	6,13427015	10,8381188	10,1843423	5,9082454	5,72065467	6,95078045
17,0826551	15,3077815	19,9158364	20,0449737	15,0428243	14,5086115	15,3580877
11,6294622	11,1676514	13,9894024	12,6775136	11,2452522	11,0085582	11,7552007
15,469969	13,8939837	15,7320175	15,5112015	13,3637545	13,345515	15,5141892
24,7918233	20,7611237	24,2400886	24,1106781	20,4533231	19,8425415	18,7847601
13,8508854	11,8562841	13,0084026	15,5677871	11,2494243	11,0773152	12,5705674
11,6780363	10,1686356	14,6290637	14,1799763	9,96580195	9,94411794	11,7889608
19,1133481	19,3655871	29,5646142	32,2263325	20,0102092	18,5925604	21,3859775
11,7309567	9,92833644	12,8101091	10,5967573	10,7856639	10,6065645	12,6538075
18,5604022	15,7108252	22,1529129	21,8416724	15,3269608	14,4150327	16,8676715
12,3917642	10,961098	15,186833	14,78509	10,1223269	9,62641551	11,7410155
19,2478093	18,2484819	23,1029489	23,4264748	19,3123296	18,1842899	16,9845623

1	0	0	1	1	1	1
2071076	2071012	2071068	2071120	2071006	2071044	2071058
15.11.87	15.01.88	15.01.88	15.01.88	15.02.88	15.02.88	15.03.88
15oct1987	NA	NA	15. Jan 00	15mar2001	15. Feb 03	15. Sep 95
breast	none	none	breast	colon	colon	breast
18,8340373	19,9948988	20,4582224	19,6075017	19,0244306	19,2073111	20,5470829
20,7413689	15,4204525	17,3677268	17,0295007	15,896944	16,5558043	17,0792118
17,4606578	18,855179	16,9873349	21,2593523	18,1292536	16,844623	18,3469756
20,9163553	19,5243733	17,99911	20,6819083	18,7956144	19,56595	19,6171885
20,0240851	14,61926	18,174672	19,6039197	16,5568757	15,6333947	17,5576509
19,443025	14,965656	17,7716978	17,3379384	15,9542277	18,4039771	15,5475584
12,9893787	10,8767441	10,7013915	11,9809534	10,5441589	10,4884707	9,46073315
16,897604	16,1426137	16,1574914	19,1305391	16,7384511	14,8135892	15,5199731
21,4377139	19,6014339	21,5807681	24,7321162	20,3797572	19,9540625	24,8668204
8,05952738	6,67008218	7,67748645	10,6688624	9,07476789	7,68818087	9,72764597
16,956862	15,5212635	16,7067705	20,5933501	18,286069	15,8947614	19,6057483
12,6695315	11,5622632	12,0002982	14,5985965	12,6317917	10,5723933	13,7007696
17,3688781	14,667578	16,7765003	17,2655203	15,888198	14,6789916	16,3176465
28,8250653	21,3399467	24,584163	25,3845823	23,2105537	22,9610001	25,33388
17,6397604	12,5944323	13,4958946	15,3991204	14,1522127	13,3747733	14,1552561
14,799618	10,9647917	13,3016341	15,2151933	12,6022388	11,7787574	13,5947944
21,5406994	20,9374215	21,1780125	24,3367271	23,6434983	18,9805768	24,4445928
16,3588095	11,749761	12,0954769	12,7616407	11,7949319	11,8227318	11,0452714
21,7798126	17,1620077	20,4073429	23,2927887	19,2536698	18,0332924	22,0167343
15,7638007	11,6081174	14,1491814	15,527023	13,2477333	12,4982074	13,898264
19,6356876	21,1153242	17,8717372	23,6646624	20,8973119	18,6974793	21,1967015

1	1	0	1	1	1	1
2071075	2071097	2071003	2071011	2071061	2071095	2071046
15.03.88	15.03.88	15.04.88	15.04.88	15.04.88	15.05.88	15.06.88
15. Feb 92	15mar2009	NA	15may1990	15mar2000	15. Apr 07	15. Nov 00
breast	lung	none	lung	lung	breast	colon
20,9105935	18,9112585	19,5259552	19,973366	21,7984694	21,581501	19,4252682
15,6463566	15,8445908	18,6780238	15,8767221	16,5830323	14,4099097	14,4956336
15,9540507	16,0713686	16,6089552	17,9806942	18,4078512	19,0037075	16,5446353
19,0889643	17,6744823	19,3343179	17,4944171	16,7961645	20,6066007	18,5192008
18,6052587	16,0392886	18,4002889	16,226225	15,7795625	17,3498963	14,0325947
15,0328759	17,7061104	19,6390873	17,030865	17,0050538	13,8519772	15,5346044
10,5407445	10,491601	10,846327	9,2283	9,16649219	10,5705074	9,5633862
17,2913538	15,4384785	15,6342169	15,174037	15,453701	16,1738394	14,2695813
23,1368715	19,5026504	21,7444944	19,7300133	20,4785117	22,0413627	16,2016411
9,24021165	7,65357519	8,7420684	9,64689267	9,33681392	8,66481563	5,84399918
18,0070574	16,2247975	17,2869258	15,204681	16,0991573	16,9480869	14,2985625
13,6984449	11,4171806	11,9227939	11,5729999	11,6889297	12,8002431	10,773169
16,0043364	15,6419143	17,3515232	15,3112166	14,824339	15,2646564	13,7951441
24,5077141	22,7099547	25,1328184	24,7107003	22,4452014	22,8626831	21,1205165
14,1488694	13,1711924	13,8066063	14,243946	13,7701963	13,5799386	11,9932715
13,1022857	11,8843677	14,6220167	12,0516007	12,3356369	11,7385519	10,0153891
29,0810185	20,3085183	24,7384087	20,286544	22,0715208	26,7147856	18,0864504
10,8853409	10,8656703	12,8789736	10,3133532	10,028063	10,6204699	10,834793
21,1686404	18,9197587	21,7833883	20,0561862	19,0302636	18,7527569	15,5551293
13,973752	12,7806588	14,8812256	13,6310903	12,835637	12,3532304	10,6718408
18,5083649	17,7309084	17,5605794	19,8927923	19,430091	22,2761209	18,5622829

1	1	1	1	1	1	0
2071024	2071072	2071071	2071023	2071036	2071079	2071034
15.08.88	15.08.88	15.10.88	15.11.88	15.11.88	15.12.88	15.02.89
15oct2006	15oct1999	15mar1999	15dec2006	15. Jan 06	15. Jul 95	NA
lung	colon	lung	lung	breast	breast	none
19,7031324	19,109837	20,7893557	19,3657351	20,801529	21,3027406	19,8129544
14,6099377	16,4824439	16,2914965	17,6135405	18,4888914	16,3962486	16,6042717
19,6804037	16,7522011	20,2575351	16,0894378	20,6717596	20,62329	17,4818408
17,271065	18,7663706	18,4601078	18,2258651	21,6742718	20,2431889	19,0250005
17,0506005	16,30741	17,7218511	16,7842096	17,0937731	18,6756507	16,7608111
15,896823	14,8196748	16,0274891	14,5148545	18,885729	15,0239989	16,7729806
9,46182368	10,0921726	10,4159789	9,68806001	9,84476047	10,7367113	10,5533267
15,7985634	15,8938098	17,5476373	15,808326	16,2815776	16,4480669	15,9627607
23,2686898	19,3700708	27,3135916	21,7298813	22,7489269	23,1444366	20,5236514
7,86335382	6,99417531	9,84033625	8,88595103	10,58027	9,29912272	8,02432
17,4472583	16,9683571	20,8738653	17,2082362	15,0895275	18,2698879	16,7806806
12,6567936	12,3022006	13,2104293	12,5417318	12,0638358	13,2125656	12,0438308
15,1099112	14,755556	15,9622092	15,8190998	15,9097004	15,6081657	15,7857395
23,0489711	23,3921001	25,7016312	23,3140359	25,8422271	24,0135938	23,069167
13,4813224	12,9022399	15,8965833	14,2622926	15,8309088	14,4056168	14,0622145
11,3922531	11,2300342	13,6672694	12,2054942	12,5573663	13,3623709	12,4886794
27,1992285	21,6817898	32,3797129	22,2486108	17,9930391	26,5322094	22,3386964
9,52185404	11,5839696	10,8334071	11,6137846	12,9849106	11,6573624	11,7947995
19,4819381	18,4990054	22,1606678	20,8872187	21,7525075	21,2995755	19,1598167
12,3970024	12,1973794	14,8056656	13,3873796	15,0418987	14,1513845	12,9938054
21,4313473	18,7163765	24,4866737	18,4742084	24,3290368	23,4154338	19,4562995

1	0	1	1	1	1	1
2071060	2071065	2071066	2071093	2071083	2071038	2071059
15.02.89	15.02.89	15.02.89	15.02.89	15.03.89	15.04.89	15.04.89
15may1988	NA	15. Apr 06	15. Sep 86	15mar2006	15. Jul 00	15mar1989
colon	none	lung	breast	lung	colon	lung
18,390868	20,809041	19,2532542	20,4756513	19,8989466	21,1750537	18,7858092
18,4176095	14,896063	14,2915415	17,4033511	15,7948386	16,184881	17,7633418
18,3168205	16,1597198	17,2509701	18,2410418	19,0144612	16,0117521	14,4338508
19,1599483	17,7449325	17,6381918	18,7520342	19,4175846	18,446265	18,2520273
18,2930334	15,490349	14,3904423	19,7846375	17,4858719	15,3594424	18,1156199
17,4477202	14,3432669	14,4812445	18,7842239	15,0513373	15,1902642	20,6843589
10,3425322	9,0077265	8,88499423	10,1455884	10,6773938	10,5151662	9,84251637
16,5142528	14,4780332	14,6735396	16,8310205	17,2702618	16,0997696	13,2154583
21,9823091	17,4913916	17,963203	22,2267258	23,2963569	20,6842116	16,8162643
11,4370285	5,61123231	6,95334255	10,5810018	9,11205721	7,21757916	9,09012677
16,8334742	14,512068	14,2150607	16,0258152	19,4973831	16,3734256	14,8509199
12,7893919	11,5719028	10,9162682	11,8558647	13,0258442	12,7187073	9,51742229
16,9548458	14,7868293	13,4680557	19,1664643	15,9906062	15,9507068	14,530327
27,9635669	20,4326686	20,8258785	26,3192277	23,9648982	22,7580042	21,5060797
15,5023209	11,6624491	11,7165393	16,0975204	14,2176623	13,7013979	13,9928349
14,0006125	11,0969375	10,0509255	14,6048632	12,8360668	11,5053592	13,3682082
25,1284005	20,7892051	21,4923055	23,3009286	30,1033771	19,7158287	16,8102167
11,6843152	11,2156157	9,0098476	12,0507616	10,9447467	11,3821804	12,3644936
19,1819301	15,9702789	15,7872962	20,9981029	21,5137501	18,4780393	17,3473694
12,4399141	10,4993758	10,2092866	13,7832825	13,9830753	12,715598	11,3763273
21,9601048	17,3808104	19,4473079	21,425761	22,3949488	17,678594	15,6058619

1	1	1	1	1	1	1
2071096	2071074	2071016	2071100	2071048	2071022	2071011
15.05.89	15.06.89	15.07.89	15.10.89	15.01.90	15.02.90	15.06.90
15. Jul 06	15dec2008	15may2006	15. Jan 99	15. Jun 05	15. Jul 07	15may1990
lung	lung	lung	breast	breast	colon	lung
17,0873185	19,4397434	19,9719814	22,8487574	19,1068186	19,8129544	19,1552573
16,1075313	14,827771	12,8909845	13,68839	13,6444937	16,6042717	17,8300899
20,3282189	13,1355892	17,5242105	19,0978405	19,9304262	17,4818408	17,3429494
19,050941	19,4608926	17,3416286	17,7613185	18,7483315	19,0250005	21,7239449
16,4608972	15,9919195	14,8482002	15,2929634	15,4188805	16,7608111	18,7342161
15,215796	15,1607215	13,1040108	13,1989151	12,7758322	16,7729806	17,2666334
9,21960232	9,97406505	8,91643542	8,83029845	9,64470211	25,6584369	10,5498359
16,0087807	15,3809331	14,5240217	15,3506176	15,8650081	15,9627607	14,1902157
22,404589	20,76314	18,0456137	17,5737528	21,3789142	20,5236514	18,5338235
9,07782406	6,93703981	6,01055353	7,62151915	6,61700733	20,5939579	9,71134839
17,5964574	16,7745089	16,4251953	14,8950755	17,4202399	16,7806806	14,0974833
12,3382118	11,8407265	11,0151943	11,7068015	12,4790934	23,8975322	10,3143323
14,6279034	14,8230517	13,4878903	13,6593381	14,340045	15,7857395	15,1345414
22,6787661	21,7130309	20,6524898	21,9082558	21,2808775	26,3475642	24,5967477
12,2250502	12,7359439	10,7394451	12,8771912	11,9538119	14,0622145	13,7020709
11,992237	11,1503758	9,82547984	10,1370825	10,7249519	25,9920543	13,8790722
27,7243506	23,5474878	25,2320812	19,9419467	24,4397707	22,3386964	19,8684745
10,9588656	9,46391816	8,41597749	10,4109236	9,78552774	25,0333136	11,8639297
20,1577414	18,4682677	16,2351737	16,4964166	17,7798023	19,1598167	18,3435499
12,6914167	11,7287999	9,54541252	10,8794949	11,0987898	25,2749002	12,5267825
22,7554661	15,9033181	21,896437	22,601929	24,0314073	19,4562995	20,1690341

1	1	1	1	1	1	1
2071004	2071067	2071027	2071001	2071028	2071002	2071086
15.12.90	15.12.90	15.01.91	15.02.91	15.02.91	15.04.91	15.06.91
15mar2000	15. Jan 04	15. Jul 02	15. Jun 07	15. Apr 04	15. Sep 00	15. Nov 99
colon	colon	breast	colon	breast	breast	breast
19,7693222	21,4135018	19,044728	17,9208855	19,6389355	19,6118422	19,5712083
15,3177358	16,1108363	15,1763809	16,5191342	13,3577632	12,9932732	13,8453463
20,2897374	16,3699324	17,2219583	16,8469308	20,2164378	15,1633033	18,3349518
17,5859865	19,3116906	18,4399971	18,9013614	17,3206563	18,1303942	19,1913285
16,3706058	14,9284495	14,8646592	15,5007326	15,7989735	12,1318329	15,6728491
14,2939367	16,3654495	14,3520735	15,2201239	14,206802	14,7825578	13,7819843
9,0427237	9,52387325	9,72246463	9,99772419	8,94527582	9,74836721	9,98519133
15,1742516	15,3472356	15,0569001	17,2386717	15,289247	14,5982163	15,844877
21,8094469	18,8643636	17,5897563	19,9051142	21,9345208	16,2362049	21,2584992
8,900589	6,86816881	6,23478202	6,4081598	7,59851692	6,6606012	7,28656127
17,7680663	14,9405162	15,8107132	16,1096434	17,1345485	15,4455241	18,2991771
12,4471584	10,5990788	11,5535357	11,8810596	12,6564566	9,57114151	12,3954372
15,0811188	14,5938912	13,8681184	14,9341922	13,9283394	13,4023466	15,0793884
23,2444123	22,5733241	21,5522979	21,446792	21,4655527	19,2915413	21,7591155
12,8376935	13,2415874	12,2110419	13,1076146	12,8065599	7,93528663	12,7546384
11,7997507	10,8468954	10,5281706	11,0725719	10,9910862	10,9246614	11,2317557
25,2326108	19,8838008	18,4844761	21,0159558	26,669544	17,3735871	28,6404834
9,16526533	11,2358293	11,1157333	11,1000393	9,19358631	10,6755319	10,0436568
20,0021646	17,1687975	15,8831328	17,485077	17,4469193	14,872425	18,3826072
12,6701497	11,947048	10,7038105	11,0078584	11,1612082	8,6865126	11,243831
21,84033	18,5903244	19,2049112	18,5348947	23,9099423	17,7738837	21,5863451

1	1	1	0	1	1	1
2071101	2071117	2071030	2071108	2071106	2071042	2071040
15.07.91	15.07.91	15.08.91	15.10.91	15.06.92	15.01.93	15.03.94
15. Jun 91	15. Apr 91	15. Jun 94	NA	15. Apr 91	15. Jun 91	15. Feb 94
lung	colon	colon	none	breast	lung	colon
18,5249982	20,7177055	19,8267706	18,3778863	19,1275549	18,5352202	19,1054869
18,4346666	15,7148198	15,116651	16,2330102	18,2315339	17,7127271	18,5802128
18,737394	18,0102558	16,2559292	15,1881622	16,7836473	18,257513	18,7262958
22,8614185	18,8608485	16,3529753	16,9636169	19,0858804	22,4335415	18,8161722
19,1199727	19,0783774	14,6364462	16,2487622	16,2459201	17,4372839	23,4931331
16,5944896	16,7881431	16,1448757	15,6038218	15,10481	17,7540534	17,9179745
10,8019104	10,9134109	7,93796854	9,29172383	11,9921335	11,3303761	12,98252
15,1827357	16,2781345	15,3099797	14,7593526	14,8484035	15,4389632	19,0837836
24,488852	23,4577301	18,0223138	18,5662345	16,7820089	18,3983007	29,7500897
9,41774416	9,80132395	7,50382147	6,73758182	6,9151852	8,51906266	13,0349273
17,7955933	19,466025	15,2380188	16,9438657	15,2357316	15,6558283	21,2889025
12,1474512	13,3382547	11,0017476	12,037092	11,7610133	11,9477625	13,6086973
16,5040279	16,2209893	14,9268695	15,0681441	14,8969602	15,6880633	20,6637885
27,6143228	25,5094767	22,491185	21,8029117	22,5740586	24,9497624	23,069167
14,8640802	14,3703312	13,0067482	10,928317	12,4494977	14,0854115	18,1393874
14,5722253	13,616586	10,5082586	11,873086	13,1073956	13,6719527	18,0882075
26,8708287	27,9090739	19,8313284	21,6901783	21,5077819	22,5434539	35,4423832
12,7708029	10,6458889	10,0846542	10,67773	13,2636251	13,4097845	14,5839998
21,356487	21,1928692	17,1067798	17,9816118	15,977189	18,0427826	27,3314081
13,5474768	13,7015497	10,9122161	11,6179716	10,3910227	11,5973873	17,9235374
22,2547663	21,3709393	18,479467	16,3157155	18,3610964	20,7739846	21,9234185

1	1	1	1	1	1	1
2071030	2071099	2071087	2071020	2071058	2071075	2071102
15.09.94	15.03.95	15.05.95	15.09.95	15.12.95	15.12.95	15.01.96
15. Jun 94	15. Nov 94	15. Jan 93	15. Aug 95	15. Sep 95	15. Feb 92	15. Jan 96
colon	lung	colon	lung	breast	breast	colon
18,9794658	17,7747412	18,1509083	19,9819131	21,4593732	21,5636072	19,5492501
19,1212523	18,8830421	17,0034565	20,5359077	16,329464	19,2456189	17,3432643
16,3967934	18,0454768	18,8058742	17,8555763	20,2844293	19,5432167	19,1890957
18,1419207	21,3140415	18,3182916	23,9297361	18,6447438	18,3696613	19,4636183
17,5649682	19,240861	16,4480211	19,6219695	18,7785337	19,4413508	18,0496898
19,3820089	19,71557	18,5889616	18,4140876	16,3746687	18,5275778	17,3704087
10,0900683	12,5613548	10,2989417	12,8097371	9,2761901	10,837773	11,4021832
15,2324847	19,9853274	16,2646333	16,0615024	16,5233823	17,672856	16,2680562
21,4401481	23,5177266	21,3436025	21,5306058	26,1066662	23,8916179	23,4483927
8,74014073	10,1489794	9,47865067	9,58164293	10,0254	12,6913304	10,1608315
15,4984647	17,5750801	16,6623485	16,0779621	19,1439535	18,001691	18,3657715
11,2795136	11,9349239	11,5957959	12,3617881	13,4658835	14,3059475	13,6803536
15,7338643	18,0517326	15,5304037	16,4677978	16,5019168	18,1786504	16,7959007
24,9643728	27,4427669	23,3547227	28,2663326	25,7686574	27,2961253	25,1978077
15,1965749	16,9488515	15,1102148	17,393229	15,5891354	16,0253876	14,918746
12,9739082	14,4826531	12,299521	15,1553649	13,7854871	14,871599	13,4186324
21,3595748	22,2459666	21,6386781	19,8502902	35,8803619	27,1925972	26,6551981
12,4054183	13,7210777	10,5641427	14,9676508	11,2884937	11,8618181	11,3076338
19,5830801	23,3669796	18,7711383	22,8824832	22,9106702	21,4662552	21,8397572
13,8426455	16,4552088	12,9452092	16,1831636	14,7337477	14,5524616	13,7829089
18,1959743	20,7981792	20,4994429	19,896817	23,2556012	22,5353411	21,6211242

1	1	1	1	1	1	1
2071013	2071052	2071056	2071100	2071071	2071094	2071019
15.11.98	15.11.98	15.01.99	15.04.99	15.05.99	15.08.99	15.10.99
15oct1998	15. Jun 98	15. Nov 98	15. Jan 99	15mar1999	15. Apr 98	15. Nov 97
breast	lung	colon	breast	lung	colon	colon
18,6480299	19,6054947	21,2484396	21,659512	17,5231958	18,4535944	19,4848504
15,4420334	16,5912172	15,7858054	13,7438223	15,6775806	16,2012719	16,4896284
16,2400387	15,9922879	17,6266907	19,6410919	17,2169193	17,3336948	18,084437
17,8254301	19,7547644	18,4269595	18,9258362	17,5214116	20,5101277	18,6692882
15,3213152	16,0025074	15,9585886	15,3216719	15,5622329	15,5296531	16,918858
17,1616229	16,7784743	17,5672689	12,2543079	15,6271235	15,7642566	17,3166707
8,58629746	11,9446069	9,49956005	9,67581725	10,0415183	11,3496126	9,82971338
14,6198949	14,5940578	17,5005712	15,1103007	14,5989769	15,1950293	16,2096655
19,3902182	17,5501267	19,8037012	18,4362172	19,7001527	19,8599322	19,049803
8,46414312	6,41649153	8,61800541	6,89115295	8,45527661	6,12694763	6,82442586
15,4451456	14,2305027	15,2935935	16,1717808	16,8888781	16,1472384	15,1116141
11,1084847	10,5181363	11,4611713	11,9396511	11,7997908	11,6511593	11,1519937
14,9043094	14,5698193	16,3252879	14,2225429	14,4819903	15,1334553	15,1879024
24,4131401	23,0637234	25,2321984	22,0163439	23,3967504	23,0639081	23,4881995
13,3688643	13,319303	13,5620369	12,1632518	12,9066279	12,449613	12,7174334
11,1619408	12,1965821	11,7317312	11,110241	12,682406	11,7363517	11,7124262
18,92814	21,4867367	22,5846423	24,2015313	22,6121243	20,5235771	21,1052219
10,8156263	11,9568818	10,8127963	10,112809	10,3309649	12,4559782	11,9987193
17,9292319	16,8361294	17,3667864	17,042937	17,8146418	16,3519072	17,8128021
11,6242492	12,1688548	11,5278619	10,8142587	11,2036697	11,3109967	11,1588421
18,6457308	18,0799191	19,4232443	22,0548443	20,2164947	19,7515842	19,9744708

1	1	1	1	1	1	1
2071047	2071079	2071004	2071061	2071086	2071010	2071055
15.10.99	15.02.00	15.05.00	15.05.00	15.05.00	15.08.00	15.03.01
15. Sep 99	15. Jul 95	15mar2000	15mar2000	15. Nov 99	15. Feb 00	15mar1999
colon	breast	colon	lung	breast	breast	colon
18,4385994	20,8152654	19,9716588	19,8034715	21,5297659	23,3652423	20,3035578
19,0485873	12,6289347	15,8219273	16,6873661	15,1322347	18,9715634	16,0115379
17,96912	16,3565932	19,0799118	19,8351136	19,3371642	20,3264174	16,6648565
18,6952193	15,8579965	17,7969834	18,3420851	17,4821969	22,8117851	18,47012
17,6087614	12,4977585	16,4895494	17,6077271	16,6460488	19,7353961	15,170725
17,5159245	10,8735036	13,3117709	18,0475918	13,6700866	22,3989121	16,1661359
10,5415506	7,89432962	9,86339695	9,2949742	9,77550018	17,7779721	10,0862655
15,6679224	12,9386688	15,7087313	17,0280658	15,6155525	17,6937707	14,8864614
20,8677422	15,0993603	23,2532477	20,147609	20,4547891	22,5442517	16,7410574
6,43099199	4,5540699	9,51664171	9,17794939	7,57477599	10,3791789	6,13050871
15,5580098	13,3605131	17,6203426	16,3702215	18,5807643	22,3151891	14,6894341
10,8739793	9,70924142	12,979583	12,9285852	12,4387635	17,0347167	11,6962962
15,5713346	11,9607098	16,5536027	16,5578286	15,8508053	22,4417666	14,9089137
23,9638584	19,447275	24,5247078	24,3431764	21,7143338	16,4578998	22,4386385
15,9406078	10,0918418	13,8262533	15,0493747	12,8644962	17,1498728	13,1320146
12,5523465	8,19363679	12,0348116	12,2481951	12,8913281	19,1391108	11,3318985
23,3701398	19,3094508	22,9579667	24,1083142	27,4283719	27,0438213	19,6750592
12,2456651	8,13714801	9,98692503	11,6627208	9,18064129	18,6696594	10,9517314
19,4085345	13,5453492	20,1183196	20,015014	18,1789539	19,2250586	16,3869743
13,0541184	8,10570077	13,1582846	13,2954994	11,6825501	15,4353615	11,0153598
20,3367785	17,7144954	21,0783236	21,7226003	21,1013549	21,2276591	18,2578942

1	1	1	1	1	1	1
2071072	2071045	2071002	2071021	2071063	2071006	2071038
15.03.01	15.04.01	15.05.01	15.05.01	15.05.01	15.07.01	15.04.02
15oct1999	15dec2000	15. Sep 00	15mar2001	15. Jul 00	15mar2001	15. Jul 00
colon	lung	breast	lung	colon	colon	colon
19,8096978	19,5458572	20,8179756	20,09239	20,0136775	18,925959	17,8477799
16,8252256	16,8531343	17,5864855	16,8728689	17,4185579	16,0211009	15,5744254
16,5812167	19,2758495	15,960032	15,9453687	17,3967943	16,523421	17,6961585
17,9150525	19,1176148	19,4393296	17,6114374	19,7092874	20,0352294	18,1422737
15,137552	17,6166087	14,4114608	18,3091183	16,9785707	14,7381437	16,477898
17,1219345	16,4458055	16,2960417	17,257173	16,7240364	15,679515	15,0672373
10,0306538	10,385382	11,4139848	10,8689852	11,0559735	10,1734456	9,11322086
15,5149036	16,0974791	16,7897917	15,6475738	17,2473085	14,9571614	14,7824118
19,2021542	25,2381265	15,7286166	22,9061744	19,2298384	18,2253694	18,8684166
6,99167868	8,38524539	7,1446197	9,88606714	7,49040786	6,28685206	8,26730232
15,8934688	17,4456666	15,4391988	17,922557	16,4667016	15,7954262	15,3025621
11,9757098	13,1129224	11,8845636	12,5477952	11,644345	11,6460729	10,8660369
16,5322439	16,5875474	15,0266815	16,4573727	15,2081304	14,9805375	14,9879518
23,3218899	25,300559	22,4681423	25,7937676	22,9449207	22,2317099	22,602103
14,7531834	16,0083515	12,8833827	15,7300203	14,7380225	13,803968	12,1357956
11,8455507	14,5375571	12,080762	14,7316861	12,2421569	10,9858095	11,5824632
19,625261	28,7121986	21,5422485	25,831701	20,4368225	20,674933	21,4771727
11,6175286	11,2267865	12,5250461	10,4388101	12,7697502	11,4148875	10,2445271
18,6279892	21,3011587	17,2016355	22,0337169	18,7339418	16,9075311	17,8644944
12,7422615	14,4492837	11,8657191	14,4954094	12,1554546	11,2729374	11,3739909
18,0260203	21,8647286	17,8251379	18,791948	19,8997483	19,1267873	20,1768094

1	1	1	1	1	1	1
2071120	2071017	2071046	2071044	2071053	2071089	2071025
15.05.02	15.10.02	15.02.03	15.03.03	15.06.03	15.10.03	15.03.04
15. Jan 00	15. Sep 02	15. Nov 00	15. Feb 03	15may2003	15. Jul 03	15. Feb 04
breast	colon	colon	colon	colon	breast	colon
18,0580536	19,7549657	16,5068882	17,8194215	19,0269665	19,0994331	28,5110469
14,7285303	15,8554235	14,054008	15,8755541	18,121619	13,9923663	18,5674236
17,9465801	17,8072995	14,6204293	16,335245	14,6340996	18,9916474	17,9959505
17,1087829	18,1255358	16,0045019	18,8743284	16,5841312	18,1612473	19,529054
14,5044615	14,9550762	12,2219923	15,2501439	17,9127301	15,2134397	15,147608
14,6805671	15,5652802	14,6627361	15,9102398	17,9826512	14,2938348	16,9722847
8,9440688	9,38730219	9,48203016	10,8098905	11,5216927	9,30803395	11,4653391
13,403002	13,6091477	12,0068547	15,4832477	17,6257492	15,3418262	15,5426036
16,8760356	16,9200127	15,5761992	17,0051989	21,4080161	19,6412433	17,8445636
5,7715495	5,91276727	4,46461466	6,28361011	8,66384816	6,01390556	6,58087982
13,6276154	15,1486146	18,8623675	16,2957405	15,7845712	16,5033185	16,5738519
9,94804828	11,2834898	14,9840466	11,8594918	11,4488628	11,2598542	12,8516195
13,8243247	14,0486465	14,6235618	14,0316062	17,5692437	14,7467171	16,1894568
21,3846487	22,0859023	12,5254816	21,5123045	23,6963457	21,762061	26,0650101
12,6149008	12,2980034	11,4997596	12,4414901	13,9368447	12,07746	12,7084734
10,4508967	10,729064	10,0030562	10,9341231	13,0425826	11,3677461	12,755922
19,1838128	19,2086769	18,752762	19,7902827	21,0761873	25,8645584	20,5333721
9,75297263	11,4491922	10,3702236	11,7331038	12,3654843	9,17737583	12,5178156
16,1383313	16,636366	12,9792569	16,2658789	21,993732	17,2169823	17,395452
10,3215213	11,121911	8,34844936	10,8551693	13,7465795	10,7330983	11,8164296
20,0040546	19,6914763	15,7794092	17,9225261	16,2869337	22,1556104	19,4148124

1	1	1	1	1	1	1
2071028	2071069	2071048	2071036	2071083	2071066	2071016
15.05.04	15.11.04	15.08.05	15.03.06	15.03.06	15.06.06	15.08.06
15. Apr 04	15. Jul 03	15. Jun 05	15. Jan 06	15mar2006	15. Apr 06	15may2006
breast	colon	breast	breast	lung	lung	lung
19,7242956	20,3756906	20,6440591	24,4275711	18,1845602	18,8683048	20,9553698
15,6517648	14,3736226	14,1729136	13,9651473	14,8562323	14,4190326	13,8687309
17,9478361	18,4145031	18,1586287	18,414667	15,9272367	20,7412961	19,9865333
17,8443744	16,5919603	18,9348801	17,931189	17,5313198	18,2490525	17,4452625
16,9391922	15,2468123	16,3865191	15,7026344	15,2985417	16,2824411	16,4205283
15,451791	13,407967	13,9111976	12,2739473	14,4194487	13,1723364	14,660888
10,1191031	8,51116736	9,4911916	8,3025183	9,46457688	10,437361	10,1284897
16,0954156	14,155519	14,4596507	14,1861769	14,8674712	15,0432258	15,7052647
20,4382046	21,6338046	18,7026928	20,300309	16,6527709	20,249384	21,8889398
7,60631553	6,55490426	6,52067217	7,02939394	6,2572425	6,77956049	7,83379672
16,6074173	17,5561734	16,0892691	16,1473379	16,2971812	16,7829111	18,0290556
11,6577255	12,0072386	10,9634921	11,7132264	11,8358666	12,0465153	12,6216878
15,9431634	16,3107639	14,5957937	15,1361988	13,7816459	14,755808	15,764472
24,102382	23,1523178	21,7178731	22,0565826	20,9619967	21,2529301	22,831696
13,4231778	14,3048989	11,8896579	12,0306218	11,8572978	12,6043519	13,8377017
13,0249734	11,0258835	11,049337	11,0656178	10,5944529	11,9272619	11,8568311
24,593628	29,5270585	22,7102852	24,3287785	21,3002091	26,6903448	26,383611
10,4693812	8,60640102	9,74713472	9,50612041	10,7556193	9,81827647	9,26547051
19,2237254	19,4079026	17,0336313	17,5858612	16,3974884	18,6117568	19,0368925
12,6179264	11,8736701	10,9041363	10,1840286	10,0028406	11,2715598	11,9327783
21,3450021	20,3476072	21,4508786	20,8535055	18,1750347	22,6086753	22,0156987

1	1	1	1	1	1	1
2071096	2071119	2071024	2071023	2071095	2071001	2071027
15.08.06	15.10.06	15.12.06	15.01.07	15.05.07	15.01.08	15.02.08
15. Jul 06	15. Sep 06	15oct2006	15dec2006	15. Apr 07	15. Jun 07	15. Jul 02
lung	colon	lung	lung	breast	colon	breast
18,380845	18,7022213	22,024865	19,949943	20,4203278	18,5363655	19,8129544
14,7988071	14,5971815	13,4031118	15,5674067	13,5892553	14,1361727	24,4409526
19,5711911	16,9331987	17,1827212	21,6140644	17,9114369	16,7656681	17,4818408
20,0068028	18,9257462	16,1982927	20,4036611	18,0006347	17,1652866	19,0250005
16,948599	14,2577555	13,4610394	18,5876741	14,7535196	13,4008878	24,6015626
14,3042726	14,7306763	15,2291508	13,1166715	13,6128417	13,4102962	25,4624613
9,96720717	10,0444097	7,30340296	12,2308688	9,32661561	8,75217069	32,1478378
15,1068254	15,1897759	14,110652	14,8652445	14,2125852	14,5341931	30,5359019
19,5374727	16,7837084	16,5748812	23,7821312	19,229018	16,1384971	20,5236514
8,20648828	5,46934747	6,49920345	9,82420944	6,64471462	4,4594546	18,7621833
17,1618271	15,2017523	13,2890541	18,5323649	16,1532225	14,1113384	31,7823463
11,8799683	11,3396941	10,4247174	13,5087101	11,1602624	10,442682	25,3606872
16,0414404	14,0142592	13,4981697	15,7315225	14,5341935	13,6155095	15,7857395
23,6334831	19,7650379	20,2570943	23,9786343	22,3019402	20,5576868	27,8983819
13,7498054	11,6611274	11,776954	13,346154	12,2849694	11,0408988	23,0354504
11,9750266	9,97386907	8,86734251	12,6115187	11,0137736	9,9824064	25,4261722
27,2079564	22,0452931	20,0918	27,6801978	24,5760237	20,4250037	22,3386964
10,6641481	10,7461201	8,35836739	12,1381055	9,30032191	9,69173421	24,5219836
19,234066	15,5433017	14,8423867	21,0149672	16,8202561	13,6261666	38,2658933
12,5841475	9,72741989	9,45830577	13,8477812	10,182168	8,58600071	22,9801068
21,6699303	18,9061626	18,2601484	23,7221974	20,6548771	18,9843831	19,4562995

1	1	1	1	1	1	1
2071067	2071103	2071008	2071050	2071080	2071112	2071081
15.02.08	15.03.08	15.05.08	15.05.08	15.05.08	15.05.08	15.09.08
15. Jan 04	15mar2007	15. Apr 08	15. Apr 08	15. Aug 06	15. Apr 08	15. Aug 08
colon	colon	colon	breast	colon	lung	breast
18,5952964	18,9311386	18,4397501	19,4961885	19,3967442	18,0245315	19,0100301
17,1972235	15,0318132	12,8753668	14,589726	15,701129	16,0026671	12,8312476
16,6705396	15,5871556	17,6990123	15,9610447	18,8738464	16,9474545	17,0627841
18,2542738	17,5573676	17,163037	18,2636793	18,5584271	21,4702085	16,9002674
16,295182	13,6328828	13,6304979	13,5886659	16,8043451	14,5094611	13,3517356
16,087493	15,1411108	13,0144044	13,7108759	14,7241912	15,6700759	12,8321322
9,14540226	9,64207619	8,67921217	8,6423927	9,73684349	10,8051919	8,39108863
14,876889	14,5971692	13,5801501	14,2988213	15,0432486	13,6037683	14,2002976
16,71249	15,3790188	18,2799186	15,5435607	21,6629303	17,6327608	15,9943345
7,11288464	5,43600193	4,87080409	4,97493852	6,86596798	5,3282829	4,54755336
15,9344658	14,0342581	14,0482301	14,0567008	17,3822067	13,5285756	13,9015237
11,4545891	10,7250515	10,3718903	10,6940291	12,2021047	9,8508236	10,4297969
15,2532007	14,2898568	13,5752492	13,5016863	15,545422	14,5663581	12,8994943
22,0134494	21,7235573	20,3528384	20,0913124	23,124885	21,6937278	18,9304387
11,305032	11,0383503	11,760568	11,452811	13,8408246	13,1903856	11,0702834
11,3566315	10,5688687	9,13293221	9,98205764	12,5453367	11,3297831	9,12298541
17,9268433	18,5158452	23,5142526	19,1086997	27,6464684	19,4589533	20,8093196
11,4664739	10,8047548	8,25828955	10,2414032	9,6668942	11,6678359	9,07859406
16,8862576	13,6874946	14,3758318	14,2555093	19,5356761	15,1268352	14,1154351
11,6865674	8,8313748	8,90600447	9,11302284	11,8992582	10,4989533	8,51202704
18,4150549	17,8056983	20,7138867	17,7949612	23,4686739	19,3613017	18,8200512

1	1	1
2071022	2071074	2071097
15.12.08	15.01.09	15.09.09
15. Jul 07	15dec2008	15mar2009
colon	lung	lung
17,9599471	19,5608764	19,4546837
12,9192538	14,5636598	15,8450651
16,0910598	17,2795157	13,1609
18,2306486	17,6339413	15,9988928
12,670878	14,4930593	13,3076438
14,4880278	14,802867	12,6699397
7,52126857	9,82965005	8,94913993
13,6751817	14,6627149	13,9085306
15,2802428	19,3746942	15,282595
4,9716479	5,19747058	4,57024931
14,3974199	16,5116864	12,6616778
10,4991663	11,063052	9,53556756
12,5457397	14,3262661	13,8204029
19,8974975	21,0841538	21,1767307
11,2805854	12,296972	10,5443367
8,81796145	11,0818549	10,6006212
18,3816943	26,2844939	19,3927419
10,0368591	9,04673862	9,0675387
14,6187089	16,3759169	13,0441911
8,69061296	10,1179788	7,95962095
18,0829453	20,2029711	15,2887325
